# Fatty acid-binding protein 5 is a functional biomarker and indicator of ferroptosis in cerebral hypoxia

**DOI:** 10.1038/s41419-024-06681-y

**Published:** 2024-04-23

**Authors:** Hao Peng, Shan Xin, Susanne Pfeiffer, Constanze Müller, Juliane Merl-Pham, Stefanie M. Hauck, Patrick N. Harter, Daniel Spitzer, Kavi Devraj, Borys Varynskyi, Thomas Arzberger, Stefan Momma, Joel A. Schick

**Affiliations:** 1 Genetics and Cellular Engineering Group, Research Unit Signaling and Translation, Helmholtz Zentrum Munich, Ingolstaedter Landstr. 1, 85764 Neuherberg, Germany; 2 Department of Genetics, Yale University School of Medicine, New Haven, CT 06520 USA; 3 Research Unit Analytical BioGeoChemistry, Helmholtz Zentrum Munich, Ingolstaedter Landstr. 1, 85764 Neuherberg, Germany; 4 Metabolomics and Proteomics Core, Helmholtz Zentrum Munich, Ingolstaedter Landstr. 1, 85764 Neuherberg, Germany; 5 Center for Neuropathology and Prion Research, Feodor-Lynen-Str. 23, 81377 Munich, Germany; 6 Institute of Neurology (Edinger Institute), Goethe University, Frankfurt am Main, Germany; 7 Department of Biological Sciences, Birla Institute of Science and Technology Pilani, Hyderabad, India; 8 Physical and Colloidal Chemistry Department, Pharmaceutical Faculty, Zaporizhzhia State Medical and Pharmaceutical University, 26 Maiakovskoho Ave., 69035 Zaporizhzhia, Ukraine; 9 Department of Psychiatry and Psychotherapy, University Hospital, Ludwig-Maximilians-University Munich, Munich, Germany

**Keywords:** Cell death, Pathogenesis

## Abstract

The progression of human degenerative and hypoxic/ischemic diseases is accompanied by widespread cell death. One death process linking iron-catalyzed reactive species with lipid peroxidation is ferroptosis, which shows hallmarks of both programmed and necrotic death in vitro. While evidence of ferroptosis in neurodegenerative disease is indicated by iron accumulation and involvement of lipids, a stable marker for ferroptosis has not been identified. Its prevalence is thus undetermined in human pathophysiology, impeding recognition of disease areas and clinical investigations with candidate drugs. Here, we identified ferroptosis marker antigens by analyzing surface protein dynamics and discovered a single protein, Fatty Acid-Binding Protein 5 (FABP5), which was stabilized at the cell surface and specifically elevated in ferroptotic cell death. Ectopic expression and lipidomics assays demonstrated that FABP5 drives redistribution of redox-sensitive lipids and ferroptosis sensitivity in a positive-feedback loop, indicating a role as a functional biomarker. Notably, immunodetection of FABP5 in mouse stroke penumbra and in hypoxic postmortem patients was distinctly associated with hypoxically damaged neurons. Retrospective cell death characterized here by the novel ferroptosis biomarker FABP5 thus provides first evidence for a long-hypothesized intrinsic ferroptosis in hypoxia and inaugurates a means for pathological detection of ferroptosis in tissue.

## Introduction

Cell death classification in pathological studies is limited by a lack of definitive markers. In contrast to apoptosis, assignment to necrotic or programmed death pathways is an exceptional challenge. Categorization has thus relied on transient markers or inhibitor efficacy in cultured cells. This is not feasible in humans, in which retrospective extrapolation has revealed a substantial contribution of non-apoptotic cell death in stroke [1], Alzheimer’s [2], Parkinson’s [3], and Huntington’s [4] disease, necessitating identification of reliable biomarkers of cell death processes in these diseases.

Cell death due to rapid, unrestrained iron-dependent catalysis of phospholipid peroxides by reactive oxygen species (ROS) is termed ferroptosis [5]. Despite extensive characterization in vitro, ferroptosis has not been conclusively demonstrated under pathological conditions in humans. Loss of the essential regulator glutathione peroxidase 4 (Gpx4) in mice revealed potential for ferroptosis in vivo [6]. Yet complete inactivation of GPX4 or loss of its essential cofactor glutathione in man is implausible, arguing that ferroptosis may be a synthetic feature of cell culture. This is supported by reports demonstrating broad ferroptosis resistance by diffusible endogenous metabolites [7–9]. Conversely, a cumulative body of evidence in favor of ferroptosis in ischemia [10–12], amyotrophic lateral sclerosis [13] and other degenerative diseases [14–18] is based on mouse models, iron accumulation [19, 20] and involvement of lipids and antioxidants in aging brains [21].

Neurodegenerative diseases are widely viewed as cell death-based diseases. Pharmacological blockers of lipid peroxidation such as α-Tocopherol, Ferrostatin-1 or Liproxstatin-1 have been effective in several disease models [17]. Additional evidence exists for Parkinson’s disease in humans, in which the iron chelator deferiprone showed substantial protection of dopaminergic neurons in the substantia nigra and concomitant improvement in motor indicators in early-stage patients [22]. This correlates to studies showing that brain iron load increases during aging [23]. Iron plays a vital role in the Fenton reaction, a process that produces ROS. During ferroptosis-regulated cell death, an excess of iron initiates lipid peroxidation when GPX4 activity is suppressed, leading to the disruption of the plasma membrane [24, 25]. Glutamate neurotoxicity results from inhibition of system xC- and downstream glutathione restriction, while ferroptosis inhibitors protect viability in glutamate-treated brain slices [5]. Thus, multiple lines of evidence point to intrinsic ferroptosis playing a central role in ischemic and neurodegenerative diseases.

Ferroptosis ensues from propagation of oxidized membrane polyunsaturated fatty acid phospholipids (PUFAs), detected by the fluorescent dyes BODIPY-C11 and Liperfluo. While these oxidized products are used specifically for ferroptosis detection in live cells in vitro [26], they are lost during tissue fixation and processing. Additional markers have been proposed such as *PTGS2* (Cyclooxygenase-2) mRNA levels in cancer [6], or malondialdehyde (MDA) and 4-hydroxynonenal (4-HNE), which are transiently formed from peroxidized lipids but unstable [27]. Feng and colleagues reported a novel antibody against human transferrin receptor 1 (TfR1) which showed an increase upon ferroptosis induction [28] but is precluded by high endogenous expression, as noted by the authors. Other candidates have been examined in the context of mouse xenografts or are not fully established for ferroptosis in vivo. Unambiguous determination of ferroptosis in man would directly enable clinicopathological assessment and therapy, while future applications enable monitoring of oncological treatments employing ferroptosis inducers having efficacy in therapy-resistant tumors [29].

Here, we identified the fatty acid-binding protein 5 (FABP5) as a key marker of early ferroptosis. As a Nurr1 target gene [30] FABP5 has multiple roles in lipid metabolism. It binds long-chain fatty acids at the cell surface, chaperones them from the cytosol and delivers them as signaling molecules to nuclear PPARβ/δ to stimulate lipogenesis [31–34]. In a process tightly linked to oxidative stress, FABP5 and HIF-1α remodel cellular phospholipids [35]. This process may be controlled by disulfide bridges exclusive to FABP5 protein, which are redox sensitive via thiol-disulfide exchange [36]. In this work, we show that FABP5 is specifically upregulated in a feedback loop that facilitates neuronal cell death in postmortem hypoxic brains, signifying with this novel marker that ferroptosis is the primary cause of their demise.

## Results

### Biomarker discovery

**Fig. 1:**
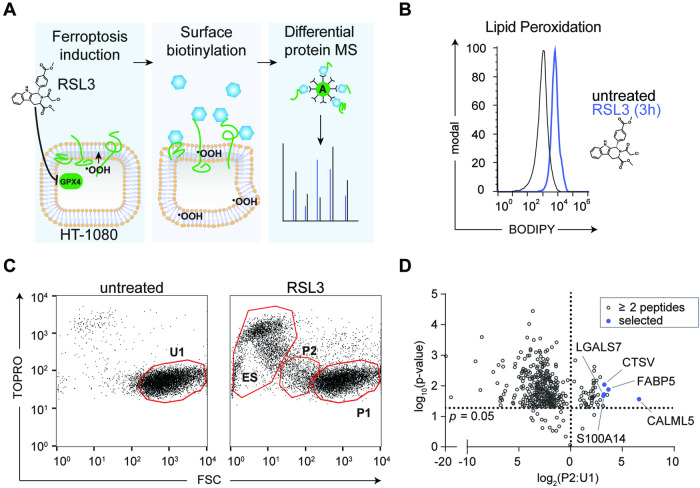
Identification of ferroptosis-specific surface proteins. **A** Experimental strategy for identifying candidate biomarkers in ferroptosis-sensitive HT-1080 cells by surface biotinylation and protein mass spectrometry. **B** Lipid peroxidation induced by RSL3 (300 nM) treatment for 3 h in HT-1080 cells. Treated and untreated cells measured by BODIPY 581/591 C11 stain (BODIPY-C11). A typical flow histogram of three independent experiments is depicted. **C** Flow cytometry of untreated cells compared to short term (3 h) RSL3 (250 nM) treated HT-1080 cells. Selected populations designated by indicated gates and ratios in Table 1 (U1, untreated; ES, end-stage ferroptosis; P1, unshifted RSL3-treated; P2, FSC-shifted RSL3-treated). **D** Volcano plot of biotinylated candidate proteins (P2) identified by mass spectrometry with ≥2 peptides in relation to control population (U1). Selected significant proteins (Students *t*-test, above dotted line, *p* < 0.05) were investigated further.

To exclude artifactual protein release induced by cell lysis in late ferroptosis, we focused on surface-exposed proteins as early candidate biomarkers. We applied surface biotinylation and antigen capture to human HT-1080 fibrosarcoma cells treated with (1S, 3R)-RSL3 (RSL3), an established model of ferroptosis, and untreated cells (Fig. 1A). RSL3 binds and inactivates the central regulator of ferroptosis, glutathione peroxidase 4 (GPX4). After 3 h of RSL3 stimulation, BODIPY-C11 indicated robust ferroptosis initiation by lipid peroxidation (Fig. 1B). Coincident with this, flow cytometry revealed three distinct populations distributed by size and membrane permeabilization (Fig. 1C), namely end-stage (ES) ferroptosis TOPRO^+^, FSC^low^ cells, TOPRO^−^, FSC^mid^ (P2), and TOPRO^−^, FSC^high^ (P1). Following lysis and capture, individual population peak proteins were quantified by mass spectrometry. We determined ratios of identified P1 and P2 peptides to uninduced (U1) samples with a cutoff of ≥2 unique peptides/protein (Fig. 1D). The highest increases in abundance were attributed to surface and secreted proteins with higher P2:U1 ratios reflecting a stronger response to RSL3 (Table 1).

**Table 1:** Protein discovery ratios and localization prediction.

Symbol	Name	P1:U1	P2:U1	*P* value	Membrane	Secreted
CALML5	Calmodulin-like protein 5	7.86	98.62	0.027	2	5
FABP5	Fatty acid-binding protein 5	1.93	12.53	0.013	5	5
CDC5L	Cell division cycle 5-like	2.06	11.52	0.019	1	0
CTSV	Cathepsin L2	1.16	9.59	0.009	3	5
LGALS7	Galectin-7	0.88	9.33	0.019	2	5
S100A14	Protein S100A14	0.79	8.96	0.021	2	5
LYZ	Lysozyme C	1.87	8.90	0.047	1	5
TXN	Thioredoxin	0.87	8.83	0.048	2	5
ANXA1	Annexin A1	1.15	8.76	0.045	5	5
S100A8	Protein S100A8	1.10	7.33	0.005	5	5

Discovery ratios for top proteins containing ≥2 identified peptides in populations in Fig. 1C. Localization based on COMPARTMENTS predicts plasma membrane and secreted likelihood (0 min–5 max).

The highest ratio was observed for Calmodulin-like 5 (CALML5; 8–99 fold increase, *p* = 0.027, Students t-test, 2-tailed), a protein linked to ferroptosis-sensitive polyunsaturated arachidonic acid metabolism, followed by FABP5 (2–12 fold, *p* = 0.013) a regulator of fatty acid homeostasis, and others including Thioredoxin protein (TXN; 1–9 fold, *p* = 0.048), which is induced and released from cells in response to oxidative stress.

### Informatic analysis

Informatic analysis and text-mining of these candidate markers revealed a network of interactive and parallel relationships in different contexts (Fig. S1a). FABP5, CALML5 and S100A8 showed the strongest relationship as top upregulated proteins in primary cutaneous amyloidosis, along with ApoE [37]. Amyloid accumulation has been shown to induce ferroptosis [38]. S100A8, TXN and ANXA1 are co-regulated pathogenic disease genes in AD [39]. Similarly, lipid-regulating enzymes ANXA1, S100A8, and FABP5 are co-regulated in a network with ApoE and APP in response to sepsis [40]. The known calcium-binding proteins (CALML5, S100A8, S100A14) modulate cancer therapeutics and survival [41, 42]. Several candidates, especially TXN, S100A8, ANXA1 and LGALS7 are strongly associated with oxygen radical processes, a key ferroptosis trigger.

Gene ontology analysis and canonical pathway mapping revealed a role in innate immunity for S100A8, FABP5, LYZ, CALML5, CTSV, ANXA1 and TXN (Fig. S1B). Further involvement in extracellular matrix, chemotaxis, and fatty acid biosynthetic processes are consistent with localization to the cell surface, while KEGG pathway analysis revealed high associations of the top candidates CALML5 and FABP5 with fatty acid metabolism, suggesting a synergistic role of CAML5 and FABP5 in regulating lipid homeostasis (Fig. S1C). Calcium-dependent synthesis/elongation of oxidation-susceptible lipids in the ER is essential for ferroptosis [43].

### Validation of biomarker candidates

**Fig. 2:**
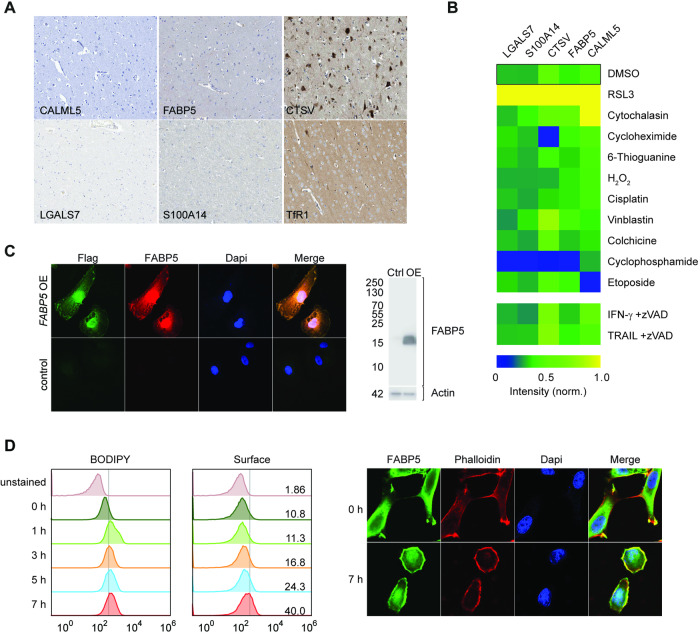
FABP5 is concentrated at cell surface during ferroptosis. **A** Immunohistochemistry staining in normal human cerebral cortex sections showing endogenous expression using antibodies against CALML5, FABP5, CTSV, LGALS7, S100A14 as well as TfR1 identified previously in [28]). The data shown here are derived from the Human Protein Atlas [68]. **B** Normalized fluorescence intensity heat map of candidate biomarker proteins following treatment with equivalent death-inducing concentrations of different pharmacological agents in HT-1080 cells. **C** Validation of FABP5 antibody specificity. *FABP5* overexpressing HT-1080 cells (*FABP5* OE) with C-terminal FLAG tag compared to control (Ctrl) by fluorescence staining and Western blot. Results observed with two antibodies (see Supplemental Reagents Table) were indistinguishable. Right panel, FABP5 expression level was detected by whole membrane Western blot in given populations. **D** Timecourse of lipid peroxidation in HT-1080 as indicated by flow cytometry analysis of Bodipy C-11 fluorescence and FABP5 cell surface staining in unpermeabilized cells and confocal images of the same cells.

We ranked top candidates by expression in normal human neural tissue and ferroptosis specificity. Another promising biomarker TfR1 indicated ferroptosis stimulation [28] but was precluded from neuropathology studies by high background expression along with CTSV, while CALML5, FABP5, LGALS7 and S100A14 showed nominal background (Figs. 2A and S1D). Next, we examined if nonspecific induction of cell death triggered expression by benchmarking antibody binding following treatment with a drug panel provoking diverse cell death mechanisms, as well as IFN-γ and TRAIL-induced necroptosis. We normalized their effective cell death-inducing concentrations to 200 nM RSL3 (Fig. S1E, F) and applied treatment to cells overnight, following by high-content antigen quantification. Robust antigen responses for four proteins (FABP5, CTSV, S100A14, and LGALS7) were observed solely with RSL3 but not with other treatments, whereas CALML5 was triggered by cytochalasin (Fig. 2B). This suggests that CALML5 upregulation occurs indiscriminately in certain types of cell death, rendering it inappropriate as a criterion for identifying markers exclusively associated with ferroptosis.

Therefore, considering its role in fatty acid metabolism and its specificity, we focused on FABP5 as the primary candidate for early ferroptosis discrimination. We validated antibody specificity against FABP5 protein (Fig. 2C) and localization in the cell, which comprised primarily of cytosolic and membrane compartments in HT-1080 cells. Upon ferroptosis induction, FABP5 concentrated at the cell surface in HT-1080 as well as in tested renal cell carcinoma lines, which are highly sensitive to ferroptosis (Figs. 2D, S2A, B). This response showed labeling up to 40% of 200 nM RSL3-treated cells by cell surface staining and was delayed compared to the burst of lipid peroxidation, indicating FABP5 is concentrated at the membrane within a few hours following ferroptosis induction. 2′-7′-Dichlorodihydrofluorescein diacetate (DCFH-DA) indicated accumulating oxidative stress that additionally could affect FABP5 redox sensitive-bonds and translocation (Fig. S2C), like the recently discovered PDRX3 [44]. Cytosolic ROS levels remain high in RSL3-induced living cells over time (Fig. S2D), which parallels FABP5 accumulation in the membrane.

**Fig. 3:**
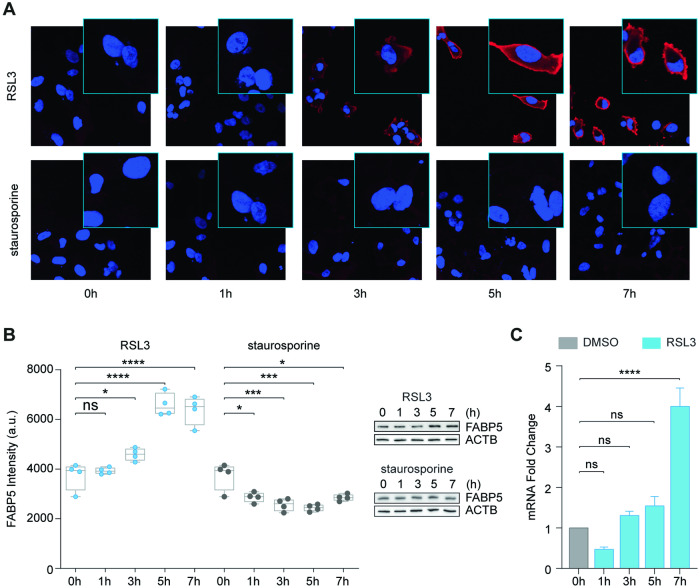
FABP5 is specifically upregulated during ferroptosis. **A** Timecourse in hours (h) of RSL3 (200 nM) or staurosporine (50 nM) induced changes in FABP5 as detected by confocal microscopy in *FABP5* OE cells. **B** High-content analysis of FABP5 in HT-1080 cells is shown as normalized mean fluorescent intensity ± SD of *n* = 4 replicate samples representative of at least three independent repetitions of the experiment in A treated with RSL3 (200 nM) or staurosporine (50 nM). Western blots show FABP5 protein detection at the same time points following treatment. **C** Relative *FABP5* expression by quantitative PCR with RSL3 (200 nM) time course. Values are shown as mean ± SD of *n* = 3 technical replicates related to untreated (0 h). Statistics were calculated using two-way ANOVA against respective control conditions.

Next, we examined expression dynamics in a timecourse by quantifying immunofluorescence at equivalent rates of cell death with 200 nM RSL3 compared to 50 nM staurosporine. FABP5 protein intensity increased significantly following RSL3 treatment, while staurosporine, an apoptosis inducer, resulted in decreased or unchanged signal, similar to the other top candidate antigens (Figs. 3A, B and S3A). Treatment with erastin resulted in a highly comparable increase in signal level and translocation (Fig. S4A). Quantitative mRNA expression dovetailed with antigen recognition in RSL3-treated cells (Figs. 3C and S3B) with a slight delay, suggesting enhanced stability or translation of FABP5 may increase signal prior to upregulated mRNA expression. As FABP5 expression and localization are strongly affected by RSL3 and erastin-treatment, our data suggest these are unique hallmarks of ferroptosis.

**Fig. 4:**
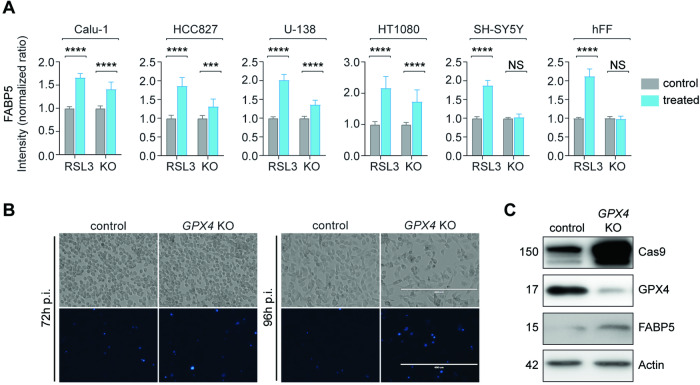
FABP5 biomarker validation in RSL3-treated and *GPX4* knockout cells. **A** FABP5 fluorescence intensity changes after RSL3 or *GPX4* knockout (KO) in cell lines of different etiologies. Calu-1, HCC827, HT-1080 and U-138MG cells after treatment with RSL3 (200 nM, 5 h), and SH-SY5Y and human fetal fibroblast (hFF) cells with RSL3 (200 nM) + ammonium ferric citrate (FAC, 250 μM) for 3 h. 2 × 10^3^ cells were assayed per condition. RSL3 final intensity is shown as mean ± SD of *n* = 3 independent experiments with three replicate wells each. Fold change at 96 h post infection with *GPX4* KO or control (empty vector) lentivirus is shown as mean ± SD of at least three independent experiments with 2 × 10^3^ cells assayed per condition in each of three replicate wells. SH-SY5Y and hFF are viable following *GPX4* KO-induced lentivirus. **B** Live cell brightfield and fluorescence images of HT-1080 viability (indicated by DAPI penetration) were taken hours post infection (p.i.) with *GPX4* KO and control virus at 72 and 96 h. Increased loss of viability was observed at 96 h, while early detection of FABP5 antigen at 72 h is shown in **C** when only a fraction of cells have died. **C** Western analysis of respective proteins in HT-1080 cells 72 h following *GPX4* ablation. Statistics were calculated using two-way ANOVA (*p < 0.05; **p < 0.01; ***p < 0.001; ****p < 0.0001; ns not significant).

We subsequently validated the candidate marker FABP5 in cell lines with diverse etiologies induced with RSL3 or genetic ablation of *GPX4*, a central regulator of ferroptosis. For the latter we chose a guide sequence that targets the key active site selenocysteine for generating Cas9-activated knockout lentivirus. Significant enrichment was seen in all RSL3-treated cultures, supported by a snapshot staining of unsynchronized early *GPX4* knockout cells with marked increases in sensitive lines, but not in SH-SY5Y cells or human fetal fibroblasts, which do not die from this treatment (Figs. 4A–C and S4B).

### FABP5 influences cellular lipid balance

**Fig. 5:**
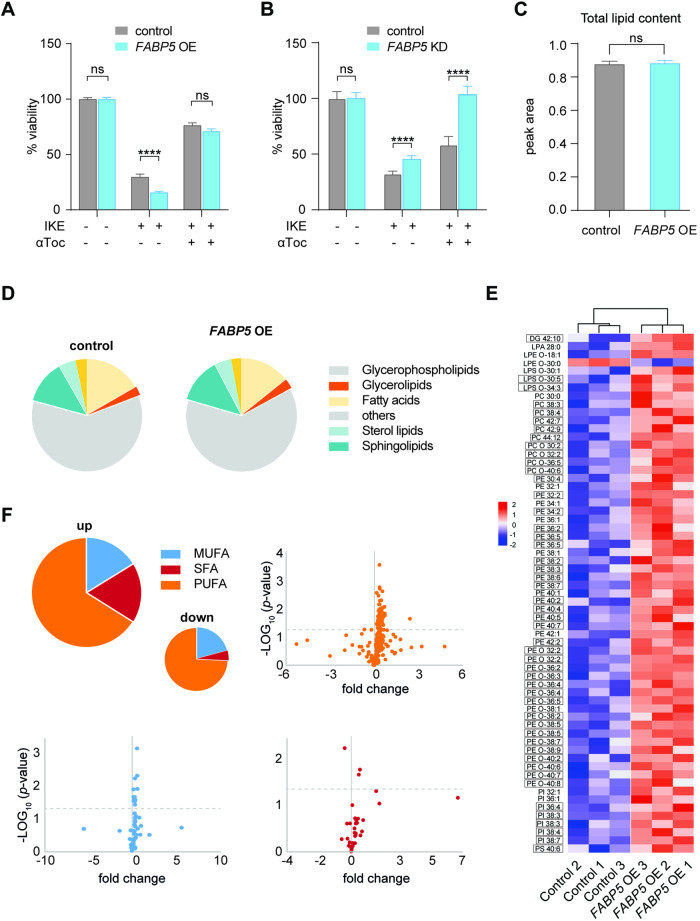
Elevated FABP5 expression induces PUFA-containing lipids and ferroptosis sensitivity. **A** Viability of *FABP5* OE compared to control HT-1080 cells treated with ferroptosis inducer IKE (1.25 µM, 16 h, two-way ANOVA (*p < 0.05; **p < 0.01; ***p < 0.001; ****p < 0.0001; ns not significant)). **B** Viability of *FABP5* knockdown (KD) compared to control HT-1080 cells treated with ferroptosis inducer IKE (1.25 µM, 16 h, two-way ANOVA (*p < 0.05; **p < 0.01; ***p < 0.001; ****p < 0.0001; ns not significant)). **C**, **D** Total lipid abundance and distribution by peak values as revealed by mass spectrometry for *FABP5* OE cells compared to control cells. No significant differences were observed in total lipid content. Class terms are derived from LIPIDMAPS [69]. **E** Lipid heatmap annotations showing z-scores of all significantly misregulated phospholipid species in *FABP5* OE and control cell samples compared with controls. Nearly all misregulated lipids are classified as PUFA-containing glycerophospholipids (boxed entries) with the number of double bonds indicated after the colon. DG diacylglycerol, LPA lysophosphatidic acid, LPE 1-acyl-sn-glycero-3-phosphoethanolamine, LPS 1-acyl-sn-glycero-3-phosphoserine, PC phosphatidylcholine, PE phosphatidylethanolamine, PE phosphatidylinositol, PS phosphatidylserine, O ether lipids (Welch t-test, *n* = 3, ns not significant). **F** Cumulative changes in each saturation class of phospholipids up- or downregulated in *FABP5* OE cells. Lipids downregulated represent a smaller fraction of the total lipid pool, as represented by the proportion of the ‘down’ pie chart. Volcano plots demonstrate fold changes of individual lipids according to saturation, the dotted line indicates significant lipids shown in (**E**). MUFA monounsaturated, SFA saturated, PUFA polyunsaturated.

Given the strong correlation of FABP5 with early ferroptosis, we hypothesized its fatty acid-binding capacity may influence death via cellular lipid dynamics. We first tested sensitivity to ferroptosis in *FABP5* OE with imidazole ketone erastin (IKE), which functions to block cystine import and glutathione production in HT-1080 cells and observed a ferroptosis sensitization in the same cells (Fig. 5A). Correspondingly, FABP5 knockdown rendered cells significantly more resistant to ferroptosis induction (Fig. 5B). FABP protein levels are generally proportional to the rate of metabolism [45], which additionally suggested that lipid storage may be affected. We investigated global lipid changes in cells ectopically expressing *FABP5* (OE) cells but could not identify differences in lipid class or total lipid content (Fig. 5C, D). Instead, among the 500 MS-detected lipids species in *FABP5*-overexpressing HT-1080 cells mainly ferroptosis-relevant long-chain PUFA-containing phospholipids were enriched (Fig. 5E). Strikingly, many contain ether-type headgroups comprising vinyl-ethers and plasmalogens including phosphatidylcholine (PC O−) and phosphatidylethanolamine (PE O−), which have been shown to drive ferroptosis sensitivity [46] (Fig. 5E, F). In comparison, monounsaturated-phospholipids are mostly unchanged while fully saturated ones show a slight increase. *FABP5* upregulation thus serves a functional role in altering the balance of saturation towards ferroptosis-sensitive lipids, which in turn depress cell viability in response to ferroptosis challenge.

### FABP5 specifically demarcates damaged neurons in mouse stroke and human hypoxia

**Fig. 6:**
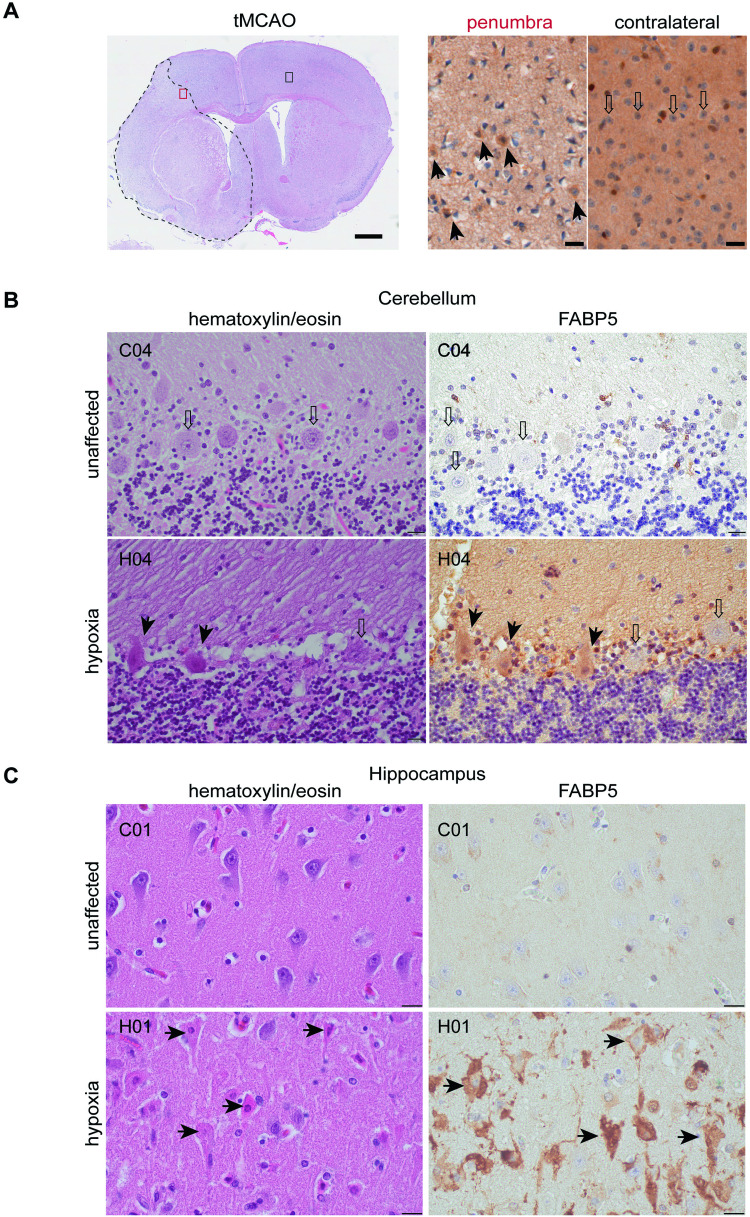
FABP5 demarcates dying neurons in cortical and hippocampal sections with hypoxic damage. **A** 15 h post-transient middle cerebral artery occlusion in mouse show evidence of ferroptosis by FABP5 staining. Left image: hematoxylin/eosin stain at the level of basal ganglia. The area of the lesion is bordered by stippled line. The red box marks an area of the penumbra, the black box an area of the corresponding healthy contralateral side. The images on the right show examples of the immunohistochemical detection of FABP5 in proximal areas. Arrows in the penumbra indicate almost exclusively neurons with partly shrunken nuclei and cytoplasmic FABP5 expression, while contralateral side neurons are healthy and unstained (open arrows). Scale bars correspond to 800 µm (left) and 20 µm (right images), respectively. **B** Human cerebellar cortex of a control case (C04) and a case with hypoxic damage (H04). (Left) Hematoxylin/eosin stains. In contrast to intact Purkinje cells (PCs; open arrows), hypoxically damaged PCs (solid arrows) are shrunken with eosinophilic cytoplasm, condensed nuclei, and an undefined nucleolus. (Right) Immunohistochemistry for FABP5 in neighboring sections, only hypoxically damaged PCs express FABP5 (solid arrows) in contrast to intact PCs (open arrows). Scale bar corresponds to 20 µm. **C** Human hippocampus of a control case (C01) and a case with hypoxic damage (H01). (Left) Hematoxylin/eosin stains. Hypoxically damaged shrunken pyramidal cells with condensed nuclei and eosinophilic cytoplasm (arrows). (Right) FABP5 immunohistochemistry in adjacent sections. In hypoxic damage almost all pyramidal cells strongly express FABP5 (arrows) in contrast to the control case without FABP5 expression. The weak brown color in the control case corresponds to lipofuscin. Scale bar corresponds to 20 µm.

Global hypoxia induction in rodents is challenging, therefore we chose to investigate FABP5 expression in an analogous region following transient middle cerebral artery occlusion (MCAO). In mice with MCA occlusion the lesion area is characterized by a loosening of the tissue structure with a severe loss of neurons and an increase of microglia. The lesion is margined by an edema (penumbra) containing neurons that mostly show signs of damage in the form of cell shrinkage and condensations of the nucleus. These alterations are already well-pronounced 15 h after reperfusion (Fig. 6A, left image). In contrast to neurons of the healthy contralateral side neurons of the penumbra show a cytoplasmic expression of FABP5 that appears more striking in neurons with morphological signs of damage (Fig.6A, right images). This region also displays a marked increase in phospho-MLKL staining indicating the presence of necroptosis (Fig. S5A) and suggesting that cell death sequelae following occlusion are of different modalities, as would be expected with an acute inflammatory response after stroke.

Next, we chose to evaluate FABP5 marker expression in vulnerable post-mortem brain regions with a relevant pathological condition. A unique group of patients who suffered cardiac arrest and reanimation, e.g., temporary oxygen deficiency with an effective reoxygenation, followed by rapid heart failure, gives a prospective window to detect a transient snapshot of dying cells. In normal cerebellum, intact Purkinje cells with clearly recognizable nucleolus and tigroid-like cytoplasm indicate healthy cells (Fig. 6B, upper left), while hypoxically damaged cells are classically defined by shrunken somata, condensed nuclei, an undefined nucleolus, and deep red cytoplasm in hematoxylin/eosin (HE) stains (Fig. 6B, lower left). Strikingly, shrunken neurons with condensed nuclei begin to express FABP5 (Fig. 6B, lower right), whereas intact Purkinje cells with clearly defined nucleoli do not express FABP5 (Fig. 6B, upper right). Similar results were observed in the hippocampus CA1 region. Hypoxically damaged pyramidal neurons (Fig. 6C, lower left) show strong FABP5 expression that corresponds to phenotypic severity (Fig. 6C, lower right). Hypoxically damaged neurons with FABP5 expression were observed for Purkinje cells, neurons of cerebellar dentate nucleus, and hippocampal pyramidal neurons across a range of affected individuals, but never in control samples, showing complete fidelity for the hypoxic condition (Table 2, Figs. S5–S7). Notably, the same regions were devoid of staining after application of an antibody against cleaved caspase-3 actively undergoing apoptosis (Fig. S8). Thus, FABP5 concurrently demarcates dying neurons, consistent with a postulated role of ferroptosis in hypoxia.

**Table 2:** Neuropathological cases with diagnoses analyzed in this study.

Case number	Diagnosis	Age at death	Period of survival after successful reanimation (in days)
H01	Hypoxic brain damage	52	3
H02	Hypoxic brain damage	59	3
H03	Hypoxic brain damage	35	7
H04	Hypoxic brain damage	32	1
H05	Hypoxic brain damage	45	2
H06	Hypoxic brain damage	81	6
C01	Control case	50	n.a.
C02	Control case	54	n.a.
C03	Control case	66	n.a.
C04	Control case	67	n.a.
C05	Control case	80	n.a.

## Discussion

Although ferroptosis has been proposed to be triggered by many physiological conditions and pathological stresses, direct confirmation of its occurrence has only been possible in cell culture experiments. Due to a lack of discriminating markers, definitive proof of ferroptosis in humans is thus far missing. In this study, we performed cell surface biotinylation and protein mass spectrometry to discover candidate ferroptosis biomarkers. Following extensive testing for specificity and fidelity, we identified FABP5 as a ferroptosis-associated biomarker highly expressed in hypoxically damaged cerebellar Purkinje and hippocampal neurons from hypoxic postmortem patients, inaugurating its use as a diagnostic biomarker and therapeutic tool in man.

FABP5 represents a key lipid binding protein that regulates the transport and metabolism of fatty acids. Dysregulated FABP5 expression has been reported under pathological states when excessive or defective cell death has occurred [47–50]. FABP5 deficiency suppresses the development of insulin resistance, diabetes mellitus and atherosclerosis [47]. In clear cell renal cell carcinoma, FABP5 binds transported fatty acids and their derivatives, thereby exerting a pro-proliferative role together with poor prognosis, while silencing FABP5 impairs of cell proliferation [48]. Consistently, FABP5 expression drives the proliferation of estrogen receptor (ER)-negative breast cancer cells on exposure to retinoic acid (RA), resulted in poor survival [51]. Overall, FABP5 expression strongly correlates with clinical characteristics. Thus, FABP5 upregulation is an indicator of ferroptosis-related lesions, in line with our result that FABP5 expression is elevated specifically after RSL3 treatment in vitro as well as highly increased in hypoxically damaged human tissue.

Definitive proof of ferroptosis at the molecular level has relied upon viability rescue with membrane-permeable antioxidants. These are believed to block propagation of lipid peroxides and terminal lapse of the cellular membrane. One limitation of the current study is that this rescue cannot be conducted without intervention and ex vivo analyses. Moreover, it is not clear whether ex vivo culture can accurately replicate the complex milieu present in brains of hypoxic patients. Certainly, intercellular signals, glia, neurotransmission as well as inflammation and cell death-derived damage associated molecular patterns (DAMPs) cumulatively affect cellular fate. As MCAO results revealed, necroptosis is also clearly present in the mouse model, however pMLKL positivity may also be due to invading immune cells. In this respect, it is noteworthy that all six patients with hypoxic damage displayed elevated FABP5 specifically in hypoxic-sensitive neuronal populations, whereas all controls lacked this increase. A larger study encompassing scored hypoxic-ischemic brain injury cases paired with MRI/CT would be able to further correlate ferroptosis with severity as well as cardiac arrest-postmortem interval. However, it is likely that ferroptosis markers are short-lived and cellular remnants following demise are scavenged by microglia [52]. Thus, quantifications of ferroptosis-positive cells are likely to vary substantially among patients and would be predicted to underestimate overall neuronal loss.

An increase in FABP-family proteins has been independently found in neurological disorders such as ischemia in primates but not attributed to a molecular cell death mechanism until now [53, 54]. A key function in lipid involvement following pathological stimuli has nevertheless been observed. In hypoxia, HIF-1α and PPARγ stimulate FABP proteins to take up fatty acids [35]. Uniquely in its family, FABP5 contains redox-sensitive disulfide bonds at the membrane [55, 56]. Under oxidative conditions, S-glutathionation of cysteine 127 drives PPAR activation and fatty acid binding [57]. Critically, PPAR has been shown to significantly increase ACSL4 expression [58], which we have shown drives an increase in long-chain polyunsaturated fatty acids, an essential ferroptosis substrate [59]. FABP5 preferentially binds long-chain saturated and polyunsaturated fatty acids [36, 60], suggesting it may directly influence their local levels. Recently, a report demonstrated translocation of peroxidized PDRX3 as a ferroptosis marker in in vitro samples, suggesting that relocalization of oxidized proteins may be a feature of ferroptosis [44]. It is thus tempting to speculate that oxidative imbalances driven by pathologic stimuli activate FABP5 to replenish PUFA-phospholipids in the membrane, with peroxidation and ferroptosis as sequelae. This would be consistent with our data showing a significant increase primarily in long PUFA-acyl chain phospholipids in cells ectopically expressing FABP5. However, FABP5 overexpression may not accurately reflect the outcome of translocation during ferroptosis, as the total cellular lipidome may not reflect local changes at the plasma membrane. Significantly changed lipids may nevertheless represent physical or downstream targets of FABP5. Whether FABP5 delivers lipids directly or via transactivation is unclear, however its localization to the membrane (Fig. 2C) favors the former hypothesis. The observed increase in FABP5 protein after five hours of stimulation may moreover suggest increased stability or translation that may act as a positive-feedback loop. Certainly, this role will be investigated more thoroughly in future pathological and molecular analyses. In summary, we demonstrate association of FABP5 with ferroptosis in human hypoxia, which will facilitate clinical utility as a hallmark biomarker in biopsy and postmortem samples to identify and monitor therapeutic interventions.

## Materials and methods

### Cell culture conditions

Cell lines used in this study: See Supplemental Reagents Table, HT-1080, Calu-1, U-138MG, HCC827, HEK 293T, SH-SY5Y and human normal fetal fibroblasts (hFF). All cell lines were maintained in DMEM (Thermo Fisher Scientific) containing 10% fetal bovine serum with exception of Calu-1. Calu-1 cells were maintained in RPMI Medium (Thermo Fisher Scientific) with 10% fetal bovine serum. 1% penicillin–streptomycin (Thermo Fisher Scientific) and 1% LGlutamine (Thermo Fisher Scientific) were supplemented in medium during growth at 37 °C in a humidified atmosphere of 5% CO_2_. HT-1080 and Calu-1 cells were maintained with 1% non-essential amino acids (Sigma).

### Cell surface biotinylation

A total of 20 × 10^6^ HT-1080 cells were seeded the day before to 20 × T75 flasks. Ferroptosis induction was performed using 250 nM RSL3 for 3 h with 10 × T75 flasks. The other half was treated with DMSO at equivalent concentration. Biotinylation of cell surface proteins was performed according to manufacturer’s directions (Thermo Fisher Pierce). Biotinylated cells were harvested using Accutase (Sigma) and flow sorted followed by continuation of the biotinylation protocol (Thermo Fisher Pierce).

### Label-free quantitative mass spectrometry in data‐dependent acquisition mode

Eluted proteins in Laemmli buffer were subjected to an on-filter digest applying a modified FASP (filter-aided sample preparation) procedure as described [61, 62]. Acidified peptide samples were measured on a Q-Exactive HF mass spectrometer (Thermo Scientific) online coupled to an Ultimate 3,000 nano-RSLC (Thermo Scientific) as described [43]. The room-temperature and the 95 °C elutions were analyzed in two batches in Progenesis QI for proteomics (Nonlinear Dynamics, part of Waters) as described [63]. MS/MS spectra were exported as Mascot generic files (mgf) and used for peptide identification with Mascot (version 2.4) using the Swissprot Human protein database (release 2017_02, 20194 sequences, 11329970 residues). Identifications were filtered for a peptide-spectrum-match false-discovery-rate <1%. Only unique peptides were considered for summed normalized protein abundance values, which were used for calculation of average ratios and a Student’s T-test resulting in the given list of fold changes and *p* values.

### DAB staining

Tissue samples were dehydrated and embedded in paraffin prior to cutting into 2- to 3-µm sections on a microtome (Leica SM2000R, Wetzlar, Germany). Clinical cases were stained using antibodies against FABP5 (Supplemental Reagents Table) on a fully automated Leica Bond III (Leica Biosystems, Germany) using the Leica Bond Polymer Refine Detection Kits (DS9800 + DS9390).

### FABP5 surface detection

Cells were treated with 200 nM RSL3 for 0, 1, 3, 5 and 7 h. After treatment, the cells were harvested and washed once with MACS buffer (D-PBS + 2 mM EDTA + 0.5% BSA) before stained with live-dead staining kit for 15 min (Invitrogen). After live-dead staining, the cells were washed once with MACS buffer, incubated with primary FABP5 antibody (Thermo Fisher Scientific) for 30 min, followed by two times MACS buffer washes and further incubation with the secondary antibody for 30 min at room temperature (Biolegend). After washing with MACS buffer two times, the cells were analyzed by flow cytometry (BD Symphony S6).

### Generation of gene overexpression and suppression cell lines

Human cDNA for *FABP5* containing a Flag tag at the C-terminus was amplified from HT-1080 cDNA and cloned into lentiviral expression vector pLVTHM (Addgene #12247) with IRES puromycin. A human *GPX4* knockout guide (See Supplemental Reagents Table) targeting Q72 proximal to the essential selenocysteine U73 (Uniprot P36969) was cloned into lentiCRISPRv2 (Addgene #52961). For *FABP5* knockdown, three guides were cloned independently into pLV hU6-sgRNA hUbC-dCas9-KRAB-T2a-Neo (modified from Addgene #71236), pooled, and co-infected. All viruses were packaged with a pantropic lentiviral 2nd generation system. Control cells were infected with pLVTHM empty or lentiCRISPRv2 empty lentivirus, respectively. After three days infection, overexpression cells were selected with corresponding drug resistance markers 3 days. Knockout cells were collected as given days after infection.

### Immunostaining *FABP5* overexpression cell line

A total of 3 × 10^3^ *FABP5* OE and control cells were seeded to a 96-well plate overnight, washed two times with PBS and fixed with 4% Formaldehyde solution (Sigma). Cells were blocked in 5% BSA Fraction V, 10% fetal bovine serum, 0.3% Triton-X in PBS for one hour then incubated in primary FABP5 antibody (Cusabio) overnight at 4 ^o^C followed by two times PBS washes and further incubation with the secondary antibody for one hour. As given in figure legends, images were taken either on an EVOS FL microscope in individual channels or Olympus FV1200 confocal microscope and merged in Adobe Photoshop. Note that all exposure settings were determined by the maximum signal in the series and these parameters applied to other time points.

### Cell viability assays

A total of 2.5 × 10^3^ cells were seeded in 96-well plates and treated overnight with the corresponding condition as indicated in figure legends. Resazurin (Sigma) was added subsequently to a final concentration of 50 µM and read out with an Envision 2104 Multilabel plate reader (PerkinElmer) at 540 nm excitation/590 nm emission after 8 h incubation. At least three wells of each condition were measured; in general, all viability is presented as percentage relative to respective control as mean ± SEM.

To establish drug toxicity baselines, cells were plated in 96-well plates for cell viability microscopy-based assays and the following day were treated as indicated in figures and legends. After 8 h treatment, 100 μl of 0.5 μM DAPI was added to each well of living cells and incubated for 10 min at room temperature under light-shielded conditions. Detection was performed using an Operetta High-Content Analysis Imaging System (HCA, PerkinElmer) using 350-nm excitation and 486-nm emission wavelengths for DAPI signal, digital phase contrast (DPC) was used for tracking all the cells. Harmony software was utilized to measure and analyze cell viability by counting the number of both DAPI positive and negative cells. Cell viability was determined by dividing the number of DAPI positive by the total number of cells and multiplying by 100. This value also represents overall cell death rate, choosing the drug concentrations IC20 (20% cell death) for analyses.

### *GPX4* knockout

*GPX4* ablation was induced using the guide ACCAACGTGGCCTCCCAGTG cloned into lenti_CRISPR v2 (Addgene #52961). The guide directly targets the sequence corresponding to the essential selenocysteine, Sec73, present in all transcripts. Any modification is predicted to produce a loss-of-function allele, even if cleavage is resolved in-frame. Lentivirus was produced, concentrated and applied to cells with an moi of >3 for analysis after 48 to 72 h.

### Western blot

Approximately 2 × 10^5^ cells were seeded in 6-well plate for each condition. Three days after infection, protein samples were isolated in 100 µl RIPA buffer and sonicated. Protein concentration was measured by BCA assay. Samples were run on a 12% SDS-PAGE gel and transferred onto PVDF membranes. Blocking was performed with 5% skim milk in TBS-T for 1 h at room temperature, then incubated in primary antibody at 4 °C overnight, washed 3× in TBS-T, and incubated with HRP-coupled secondary antibodies for one hour at room temperature. After three washes with TBS-T the signal detection was performed by ECL (Bio-Rad) according to the manufacturer instructions. Timecourse samples were treated with 200 nM RSL3, 50 nM staurosporine 0 to 7 h, or 600 nM IKE 0 to 8 h as well as the confocal images.

### High-content immunofluorescence intensity assay

For ferroptosis and cell death induction, 5 × 10^3^ cells were seeded in 96-well plates overnight. Culture medium was replaced with 100 µl of medium containing 200 nM RSL3 or 50 nM staurosporine for given timepoints in quadruplicate, respectively, for antibodies given in the Supplemental Reagents Table. Immunofluorescence intensity assays were performed on Operetta (PerkinElmer) using DAPI to identify primary objects with a Cy3 secondary antibody (Dianova, 1:500) for intensity measurements.

### Drug panel assay

HT-1080 cells were seeded one day before treatment at a density of 5 × 10^3^ cells/well on 96-well plates and maintained for 24 h. Next, 10 μl medium was added containing different concentrations of chemicals below per well to make the final concentrations below: Cytochalasin (Sigma, 0.015 mg/ml), Cycloheximide (CHX Merck, 12.5 µg/ml), H_2_O_2_ (Sigma, 0.15%), Vinblastine (Sigma, 0.15 mg/ml), 6-Thioguanine, 6-TG (Sigma, 10 mg/ml), RSL3 (Stockwell lab, 50 nM), Etoposide (J&K, 100 mg/ml), Colchicine (Serva, 3.75 mg/ml), Cyclophosphamide (CP, J&K, 31.25 mg/ml), and Cisplatin (Sigma, 62.5 mg/ml). Necroptosis was induced with IFN-γ (100 ng/mL) or TRAIL (20 ng/mL) + zVAD (10 μM) and rescued with Necrostatin-1 (10 μM). After 8 h treatment with the respective drugs or 16 h for necroptosis, cells were fixed with 4% formaldehyde solution (Sigma) then used for immunofluorescence intensity assays.

### Lipid peroxidation detection by flow cytometry

Cells were seeded to 70% confluency in 6-well plates. The next day, 300 nM RSL3 was added for 3 h. Subsequently, 2 µM BODIPY 581/591 C11 (Thermo Fisher Scientific) were added for 30 min incubation. Cells were harvested for analysis with an Attune acoustic flow cytometer (Applied Biosystems). 3 × 10^4^ events per condition were collected from the BL-1 channel (excited by 488 nm laser). Each experiment was repeated at least three times and representative results are shown.

### Animals

#### Animal selection and housing

The experimental procedures involved adult male mice of the wild-type C57BL/6J strain, aged between 10 and 12 weeks, obtained from Charles River Laboratories (Sulzfeld, Germany). The mice were housed in cages, with three to five animals per cage, in a controlled facility that maintained specific-pathogen-free (SPF) conditions. Animals were sacrificed by cervical dislocation under deep isoflurane anesthesia. The use of animals in the study was approved by the local governmental authorities (approval number FK/1052, Regierungspraesidium Darmstadt, Germany). All procedures involving animals adhered strictly to the guidelines set forth in the German Protection of Animals Act. Additionally, the study followed the ARRIVE (animal research: reporting of in vivo experiments) guidelines and recommendations outlined in the Guide for Care and Use of Laboratory Animals by the National Institutes of Health.

#### Mouse model of transient middle cerebral artery occlusion

Transient middle cerebral artery occlusion (tMCAO) surgeries were conducted according to the previously described protocol [64]. Initially, the mice were anesthetized with 0.1 mg/kg buprenorphine (Temgesic; Essex Pharma, Munich, Germany) and 1.5% isoflurane, while maintaining spontaneous respiration. Surgical exposure of the extracranial arteries on the right side, including the common carotid artery (CCA), external carotid artery (ECA), internal carotid artery (ICA), and pterygopalatine artery (PPA), was then performed. The ECA and proximal trunk of the CCA were ligated using a 6–0 silk suture, and a microclip was placed on the PPA. Subsequently, a small incision was made in the CCA using microscissors, followed by the insertion of a standardized monofilament (Doccol Corporation, #602256PK10) into the ICA. The filament was advanced until resistance was felt, thereby occluding the proximal middle cerebral artery (MCA). To prevent repositioning, the occluding filament in the CCA was tied. After 60 min, the filament was withdrawn to allow reperfusion of the ischemic hemisphere. Post-surgery, the animals were administered 0.1 mg/kg buprenorphine every 8 h for pain relief and were allowed to recover on a thermal blanket.

### Informatic analyses

Literature relationships between identified proteins and keywords “ROS”, “reactive oxygen species” or “oxidative stress” were conducted using Chilibot [65]. Gene ontology (GO Term) associations were determined by GSEA [66], while individual gene KEGG scores were calculated by ARCHS4 [67].

### Statistical analyses

Cells were treated in at least three separate experiments on different days and analyzed according to conditions indicated in the figure and unless otherwise specified. All statistical analyses were performed by using Prism (GraphPad Software). If not stated otherwise, significance was determined using two-way ANOVA against respective control conditions (*p < 0.05; **p < 0.01; ***p < 0.001; **** p < 0.0001; ns = not significant) ±SEM.

### Supplementary information

Supplemental materials

## Data Availability

All data presented in the manuscript are available in the supplemental files or are available from the corresponding author on request.
