# Supplementary material for: Fatty acid-binding protein 5 is a functional biomarker and indicator of ferroptosis in cerebral hypoxia
Source: Cell Death Dis. 2024 Apr 23;15(4):286. doi: 10.1038/s41419-024-06681-y (PMC11039673; doi:10.1038/s41419-024-06681-y)
Supplement: Supplementary file 1 — Supplemental materials [file 41419_2024_6681_MOESM1_ESM.pdf]

Supplementary Figure Legends

Fig. S1 Candidate biomarker expression and specificity

A Interrelatedness of top 10 candidate markers. Arc widths indicate common literature appearances, protein dot sizes represent ROS involvement

B GSEA GO-term and canonical pathways, bar size is 1/(False Discovery Rate q value)

C KEGG pathway z-scores (standard deviations from the mean) are shown for top candidates CALML5 and FABP5

D Expression heat map of five top candidates in tissue obtained from BioGPS 1 database. Expression levels are shown as colors and range is arbitrary microarray index from pooled simultaneous experiments

E Toxicity heat map of cells treated with gradient concentrations of different drugs, RSL3, Cytochalasin, Cycloheximide (CHX), 6-Thioguanine (6-TG), Hydrogen peroxide (H_2O_2), Cisplatin, Vinblastine, Colchicine, Cyclophosphamide (CP) and Etoposide. Boxed values were used as treatment concentrations in Fig. 2b to achieve 70-80% viability equivalent to 200 nM RSL3. See Materials and Methods for concentrations

F Induction of necroptotic cell death in HT-1080 cells IFN-γ (100 ng/mL) or TRAIL (20 ng/mL) +zVAD (10 μM) pan-caspase inhibitor and rescued with Necrostatin-1 (10 μM).

Fig. S2 FABP5 cell surface detection in renal adenocarcinoma, total ROS, and viability assays

A Surface detection of FABP5. Cells were treated with 200 nM RSL3 for indicated timepoints then harvested for antibody staining and flow cytometry analysis with gates set strictly for living cells by nuclear stain exclusion. Values for each timepoint are percentages of FABP5-labelled living cells. Antibody control is without primary antibody.

B Lethality triggered by RSL3-treatment in adenocarcinoma lines. Ferroptosis rescue is demonstrated by addition of alpha-tocopherol (aToc)

C Flow cytometry analysis of DCFH-DA (25 μM) with gates set strictly for living cells over time treated with RSL3 as above

D Flow dot plots of viable cells treated with RSL3 (200 nM) over 7 h incubation showing fraction of viable cells

Fig. S3 Candidate marker specificity

A High content analysis of normalized mean fluorescence intensity of RSL3 (200 nM) or staurosporine (50nM) treated HT-1080 cells induced changes in FABP5, CALML5, CTSV, S100A14 and LGALS7 stained cells over the time of treatment give in hours (h). Cellular localization and intensity of each condition at each time point in right panels are shown. Intensity is shown as mean ± SD of $n = 4$ replicate samples representative of at least three independent repetitions of the experiment with similar results

B Relative gene expression by qPCR (mRNA fold change) of candidates. Values shown are mean ± SD of $n = 3$ technical replicates related to untreated (0 h).

Two-way ANOVA against respective control conditions (* $p < 0.05$; ** $p < 0.01$; *** $p < 0.001$; **** $p < 0.0001$; ns = not significant)

Fig. S4 Erastin timecourse and *GPX4* knockouts

A Timecourse in hours (h) of erastin (600 nM) induced changes in FABP5 expression as detected by confocal microscopy in *FABP5* OE cells and Western blot.

B Cell density and FABP5 fluorescence intensity changes 72 h post-infection *GPX4* knockout (KO) or control (scrambled guide) in cell lines of different etiologies corresponding to Fig. 4A.

Fig. S5 Necroptosis detection in stroke and FABP5 detection in cerebellar cortex of control and hypoxic cases

A 15h post-transient middle cerebral artery occlusion in mouse show evidence of necroptosis by phospho-MLKL staining. The left box marks the penumbra, the right box is corresponding area on the healthy contralateral side. The images on the right show examples of the immunohistochemical detection of phospho-MLKL in nuclei in ischemic-proximal areas. Scale bars correspond to 20µm.

B (Left) hematoxylin/eosin stains (HE). (Right) FABP5 stains. In contrast to the large Purkinje cells (PCs) of control case C02 the PCs of hypoxic case H06 are shrunken, the cytoplasm is deep eosinophilic, and the nuclei are condensed, all signs of hypoxic damage. In consecutive 2µm thick paraffin sections, the hypoxically damaged PCs express FABP5, but not the PCs of the control case.

In directly adjacent 2µm thick paraffin sections of case H04, the same PCs are cut twice. Triangle and asterisk mark the corresponding PCs. The hypoxically damaged PC in HE (asterisk) is shrunken, its

cytoplasm is eosinophilic, and its nucleus condensed. A neighbouring PC (open triangle) is still well-preserved. In the consecutive section only the hypoxically damaged PC expresses FABP5 (asterisk) in contrast to the well-preserved one (open triangle). Scale bars correspond to 20µm in all pictures

Fig. S6 Cerebellar dentate nucleus of a control case and one with severe hypoxic damage

(Left) hematoxylin/eosin stains (HE). (Right) FABP5 stains. In HE of hypoxic case H03, large neurons of the dentate nucleus show typical signs of hypoxic damage with eosinophilic cytoplasm and shrunken or practically absent nucleoli (examples are indicated by arrows). Nuclei und nucleoli are well-defined in control case C05. Note that the number of glial cells in H03 is highly increased compared to control case C05.

In H03, not only hypoxically damaged neurons (arrows) express FABP5 but also glial cells lying in between, which primarily correspond to activated microglial cells. In control case C05 there is no FABP5 expression; the brownish color of the neuronal cytoplasm represents lipofuscin. Scale bars correspond to 20µm in all pictures

Fig. S7 Pyramidal neurons of hippocampal CA1/CA2 transition regions of a control case and two cases with hypoxic damage identified by FABP5

(Left) hematoxylin/eosin stains (HE). (Right) FABP5 stains. Hippocampal pyramidal cells of hypoxic cases H05 and H02 demonstrate classic signs of damage in HE stainings with cell shrinkage, eosinophilic cytoplasm and condensed nuclei. Those without discernible nucleolus also express, albeit weakly, FABP5 in contrast to well-preserved pyramidal cells of control case C03. The brownish color in the cytoplasm in the FABP5 staining of C03 corresponds to lipofuscin. Scale bars correspond to 20µm in all pictures

Fig. S8 Cleaved caspase-3 staining in dying neurons in cortical and hippocampal sections with hypoxic damage

A Cerebellar cortex of a control case (C03) and a case with hypoxic damage (H06). Absence of immunohistological signal against cleaved caspase-3 in two different areas corresponding to the regions shown in Fig. 6. Scale bar corresponds to 20µm

B Hippocampus of a control case (C03) and a case with hypoxic damage (H01). Cleaved caspase-3 signal is absent in two different areas corresponding to the regions shown in Fig. 6. Scale bar corresponds to 20µm

Fig S1

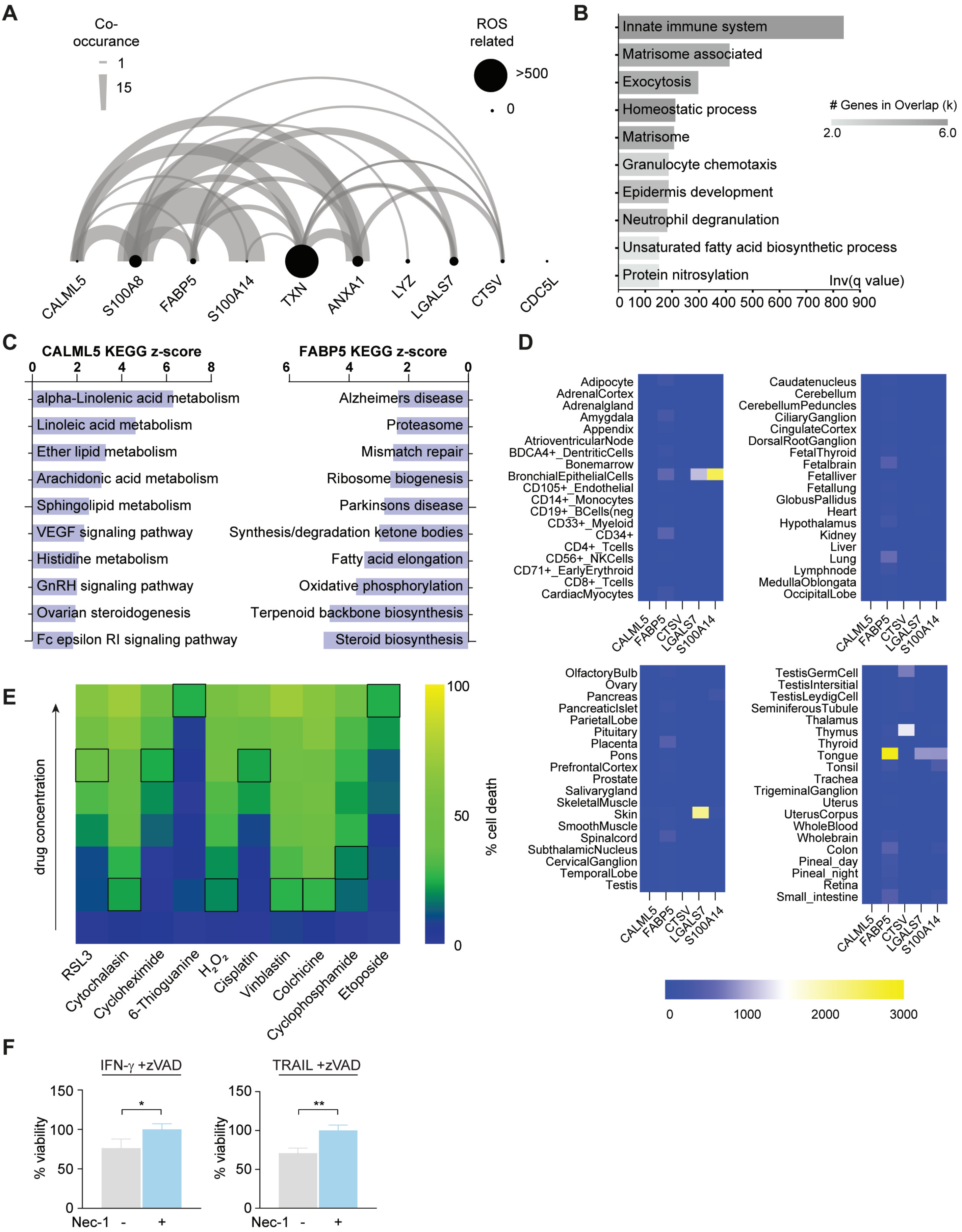
A
Co-occurance
1
15
ROS related
>500
0
CALML5
S100A8
FABP5
S100A14
TXN
ANXA1
LYZ
LGALS7
CTSV
CDC5L
B
Innate immune system
Matrisome associated
Exocytosis
Homeostatic process
Matrisome
Granulocyte chemotaxis
Epidermis development
Neutrophil degranulation
Unsaturated fatty acid biosynthetic process
Protein nitrosylation
Genes in Overlap (k)
2.0
6.0
Inv(q value)
0 100 200 300 400 500 600 700 800 900
C
CALML5 KEGG z-score
0 2 4 6 8
alpha-Linolenic acid metabolism
Linoleic acid metabolism
Ether lipid metabolism
Arachidonic acid metabolism
Sphingolipid metabolism
VEGF signaling pathway
Histidine metabolism
GnRH signaling pathway
Ovarian steroidogenesis
Fc epsilon RI signaling pathway
FABP5 KEGG z-score
6 4 2 0
Alzheimers disease
Proteasome
Mismatch repair
Ribosome biogenesis
Parkinsons disease
Synthesis/degradation ketone bodies
Fatty acid elongation
Oxidative phosphorylation
Terpenoid backbone biosynthesis
Steroid biosynthesis
D
Adipocyte
AdrenalCortex
Adrenalgland
Amygdala
Appendix
AtrioventricularNode
BDCA4+_DentriticCells
Bonemarrow
BronchialEpithelialCells
CD105+_Endothelial
CD14+_Monocytes
CD19+_BCells(neg
CD33+_Myeloid
CD34+
CD4+_Tcells
CD56+_NKCells
CD71+_EarlyErythroid
CD8+_Tcells
CardiacMyocytes
Caudatenucleus
Cerebellum
CerebellumPeduncles
CiliaryGanglion
CingulateCortex
DorsalRootGanglion
FetalThyroid
Fetalbrain
Fetalliver
Fetallung
GlobusPallidus
Heart
Hypothalamus
Kidney
Liver
Lung
Lymphnode
MedullaOblongata
OccipitalLobe
OlfactoryBulb
Ovary
Pancreas
PancreaticIslet
ParietalLobe
Pituitary
Placenta
Pons
PrefrontalCortex
Prostate
Salivarygland
SkeletalMuscle
Skin
SmoothMuscle
Spinalcord
SubthalamicNucleus
CervicalGanglion
TemporalLobe
Testis
TestisGermCell
TestisIntersitial
TestisLeydigCell
SeminiferousTubule
Thalamus
Thymus
Thyroid
Tongue
Tonsil
Trachea
TrigeminalGanglion
Uterus
UterusCorpus
WholeBlood
Wholebrain
Colon
Pineal_day
Pineal_night
Retina
Small_intestine
CALML5
FABP5
CTSV
LGALS7
S100A14
0
1000
2000
3000
E
drug concentration
100
50
0
% cell death
RSL3
Cytochalasin
Cycloheximide
6-Thioguanine
H2O2
Cisplatin
Vinblastin
Colchicine
Cyclophosphamide
Etoposide
F
IFN-γ +zVAD
TRAIL +zVAD
150
100
50
0
% viability
*
**
Nec-1
-
+

Fig S2

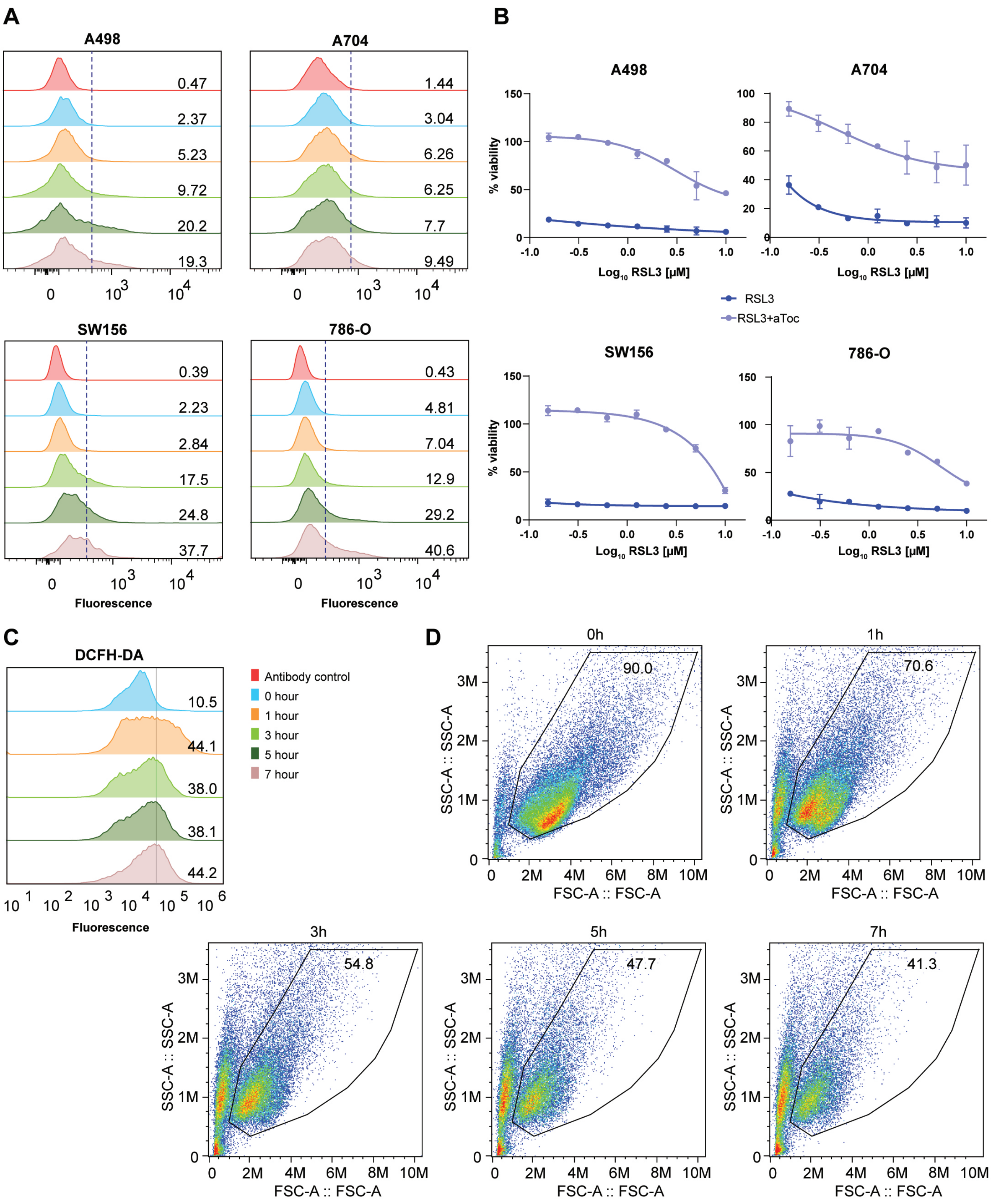

A
A498
0.47
2.37
5.23
9.72
20.2
19.3
A704
1.44
3.04
6.26
6.25
7.7
9.49
SW156
0.39
2.23
2.84
17.5
24.8
37.7
786-O
0.43
4.81
7.04
12.9
29.2
40.6
Fluorescence
B
A498
A704
SW156
786-O
% viability
Log10 RSL3 [μM]
RSL3
RSL3+aToc
C
DCFH-DA
10.5
44.1
38.0
38.1
44.2
Fluorescence
Antibody control
0 hour
1 hour
3 hour
5 hour
7 hour
D
0h
90.0
1h
70.6
3h
54.8
5h
47.7
7h
41.3
SSC-A :: SSC-A
FSC-A :: FSC-A

Fig S3 A

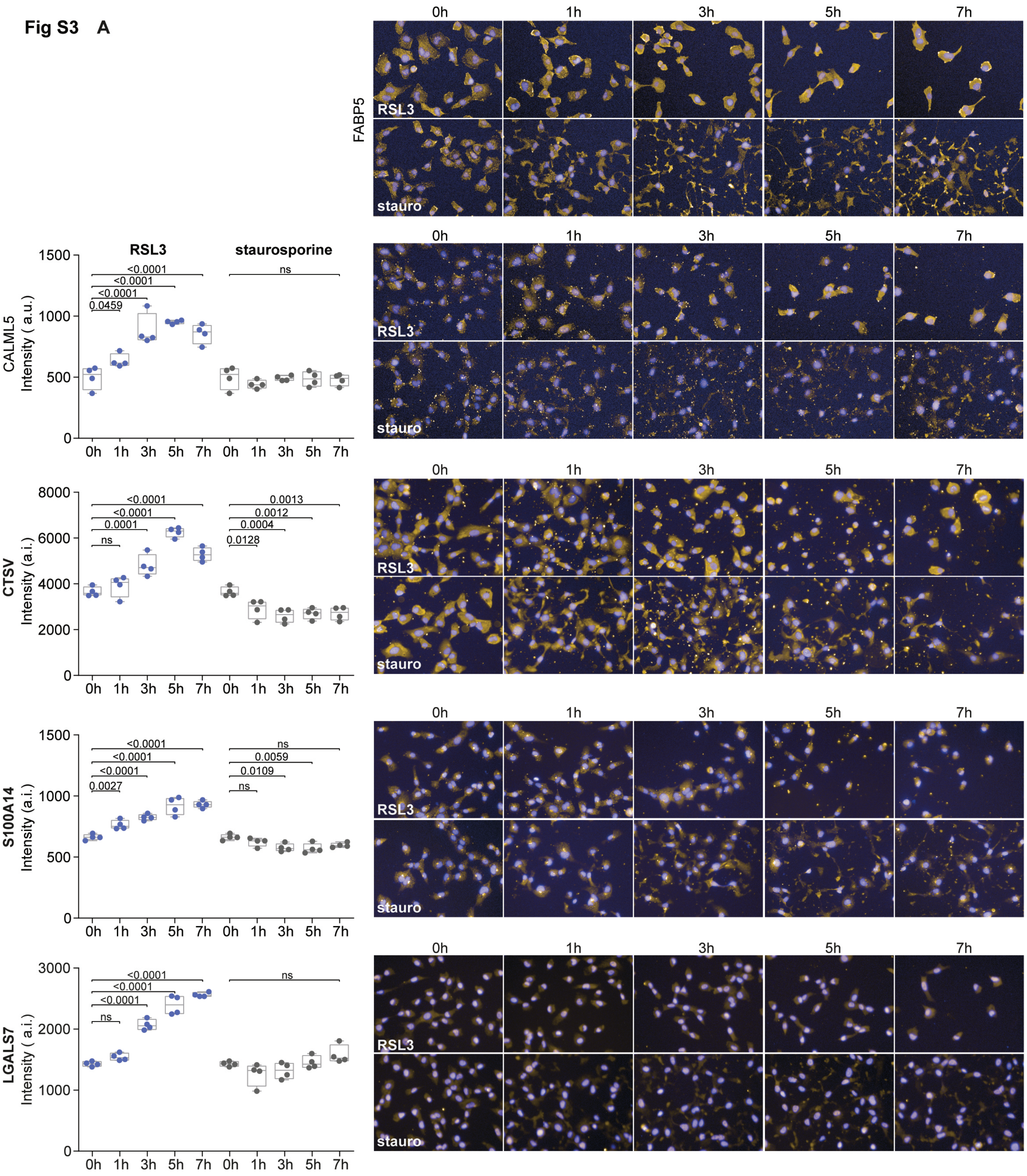
0h
1h
3h
5h
7h
FABP5
RSL3
stauro
RSL3
staurosporine
CALML5
Intensity (a.u.)
<0.0001
0.0459
ns
CTSV
Intensity (a.i.)
0.0001
0.0013
0.0012
0.0004
0.0128
S100A14
0.0027
0.0059
0.0109
LGALS7
Intensity (a.i.)

B

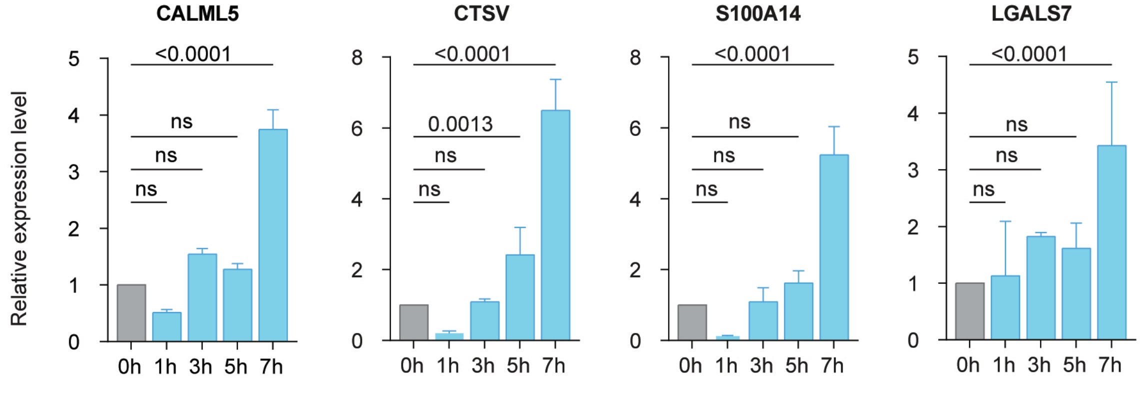
CALML5
CTSV
S100A14
LGALS7
Relative expression level
<0.0001
ns
0.0013
0h
1h
3h
5h
7h

Fig S4

A

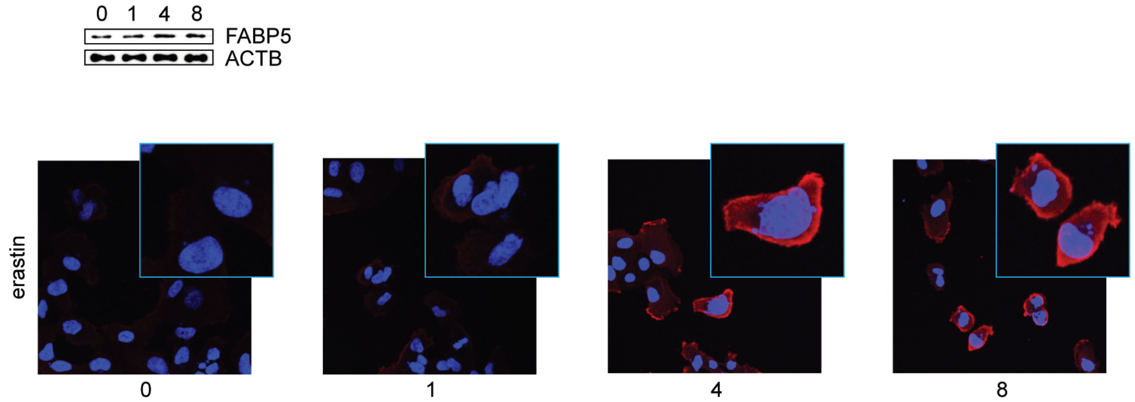
0 1 4 8
FABP5
ACTB
erastin
0
1
4
8

B

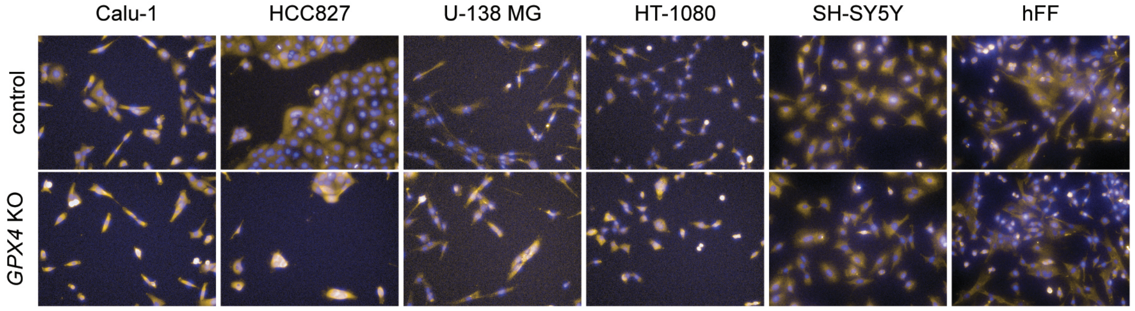
Calu-1
HCC827
U-138 MG
HT-1080
SH-SY5Y
hFF
control
GPX4 KO

Fig S5

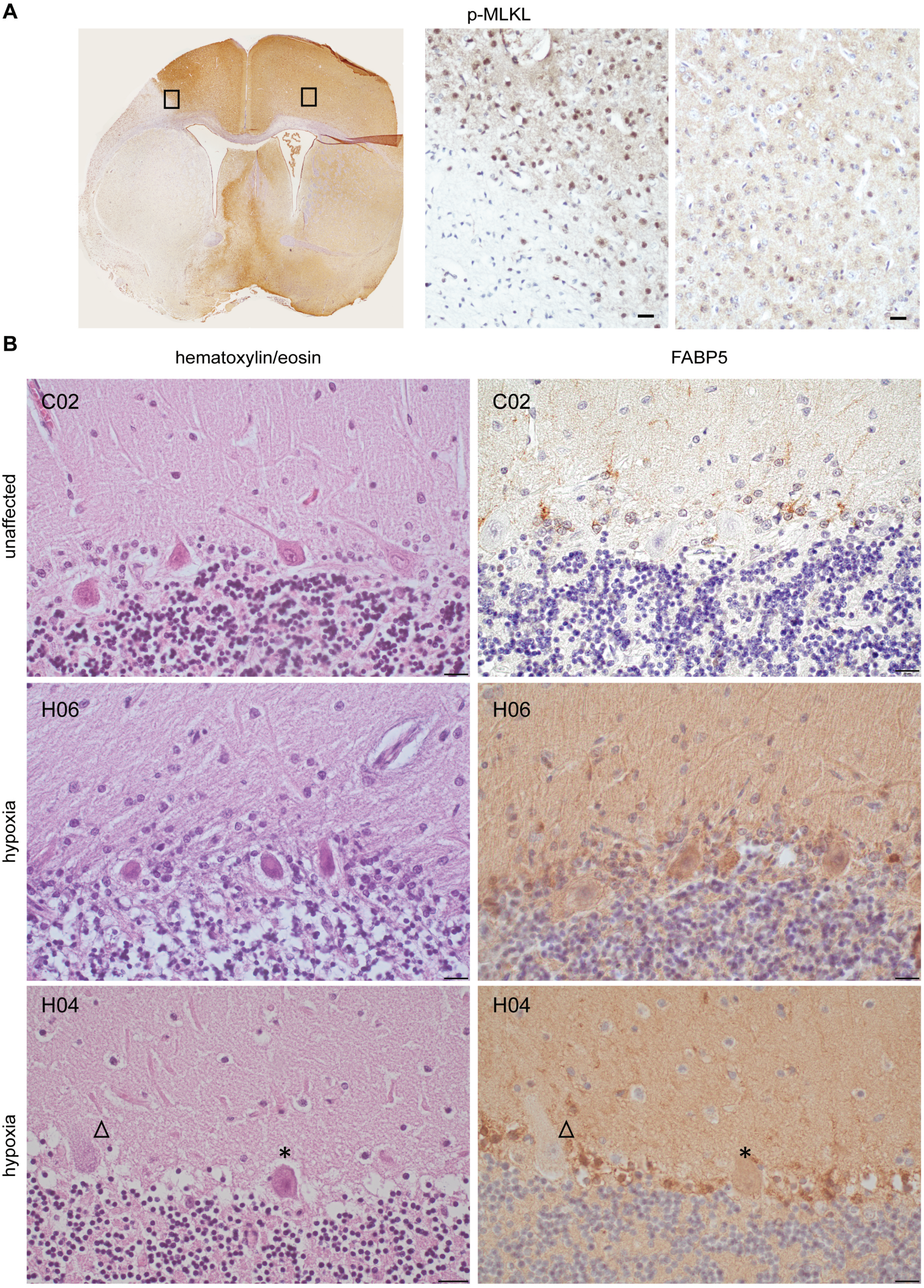
A
p-MLKL
B
hematoxylin/eosin
FABP5
unaffected
hypoxia
hypoxia
C02
C02
H06
H06
H04
H04
△
*
△
*

Fig S6

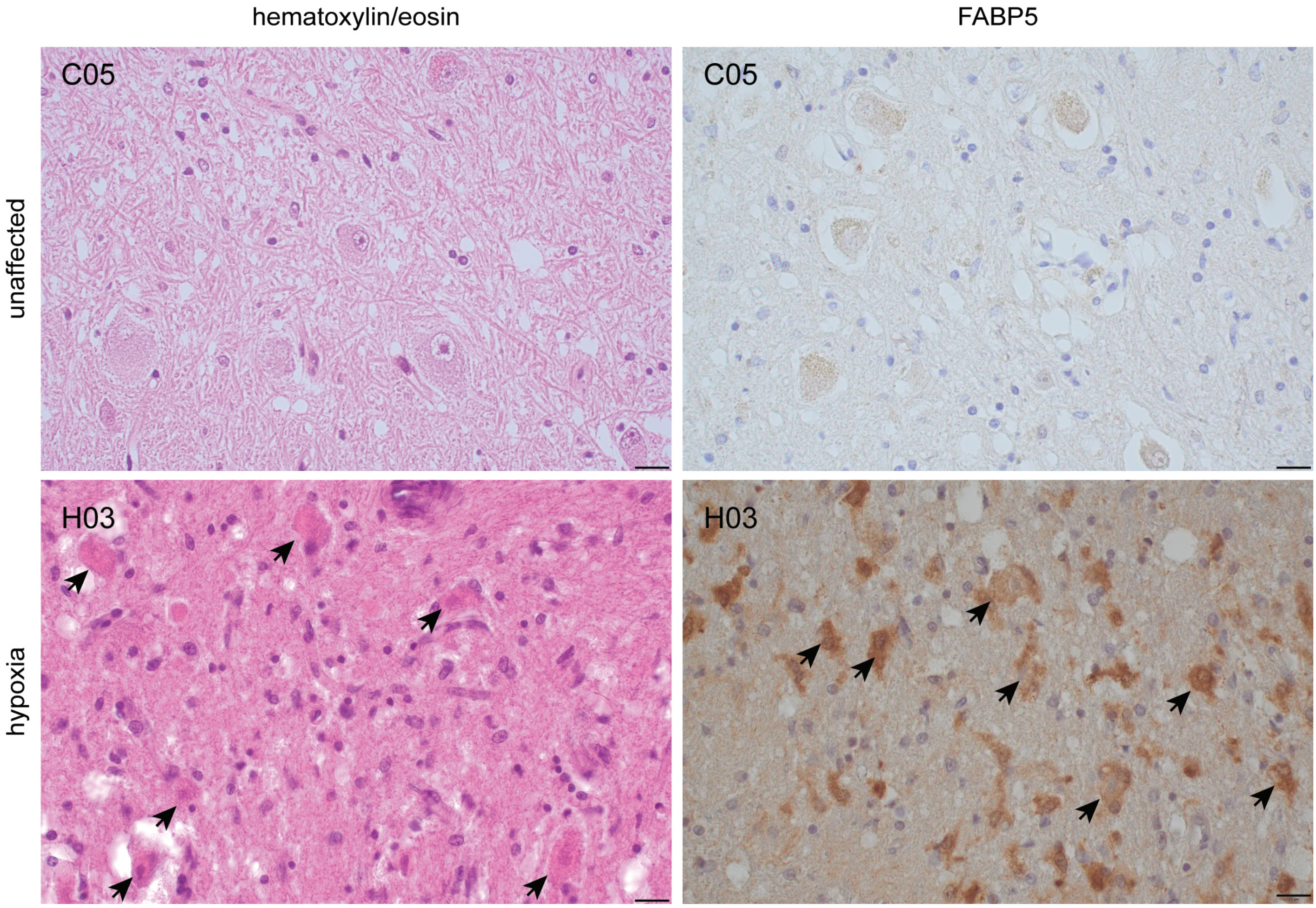
hematoxylin/eosin
FABP5
unaffected
hypoxia
C05
C05
H03
H03

Fig S7

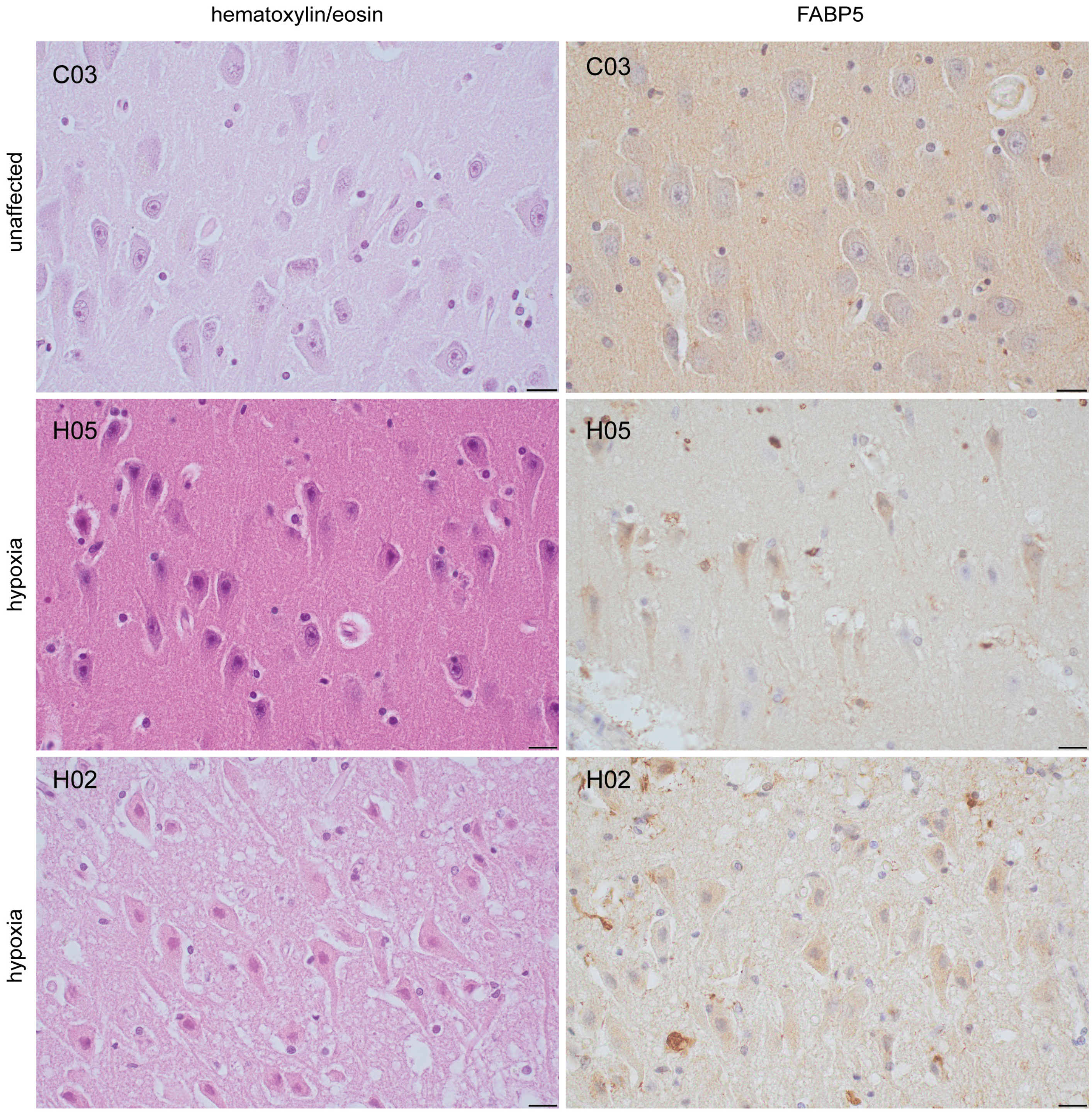
hematoxylin/eosin
FABP5
unaffected
hypoxia
hypoxia
C03
C03
H05
H05
H02
H02

Fig S8

A

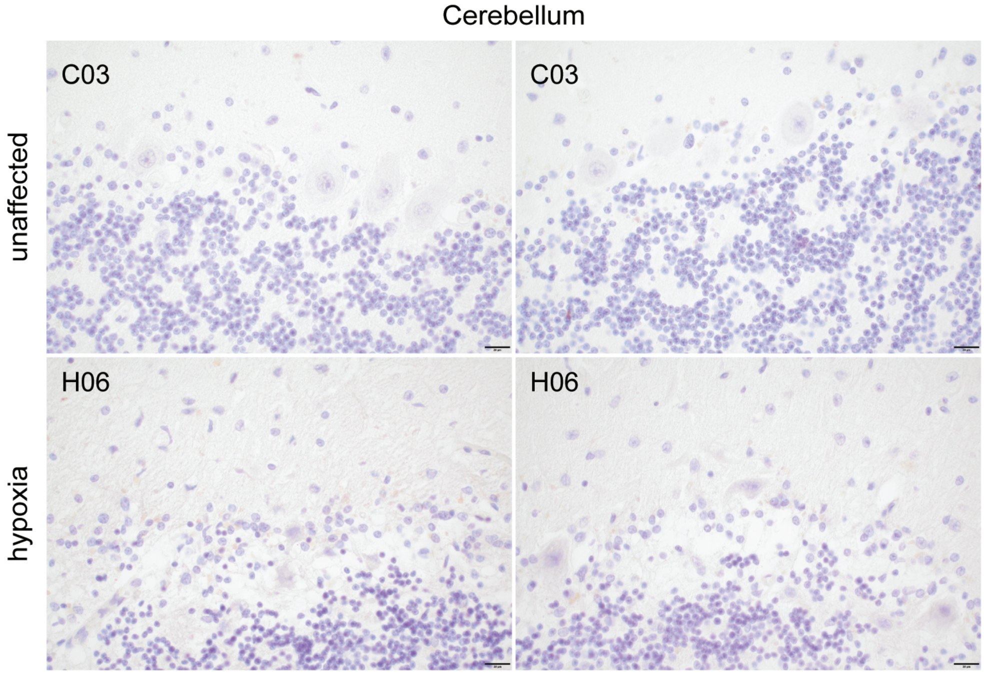
Cerebellum
unaffected
hypoxia
C03
C03
H06
H06

B

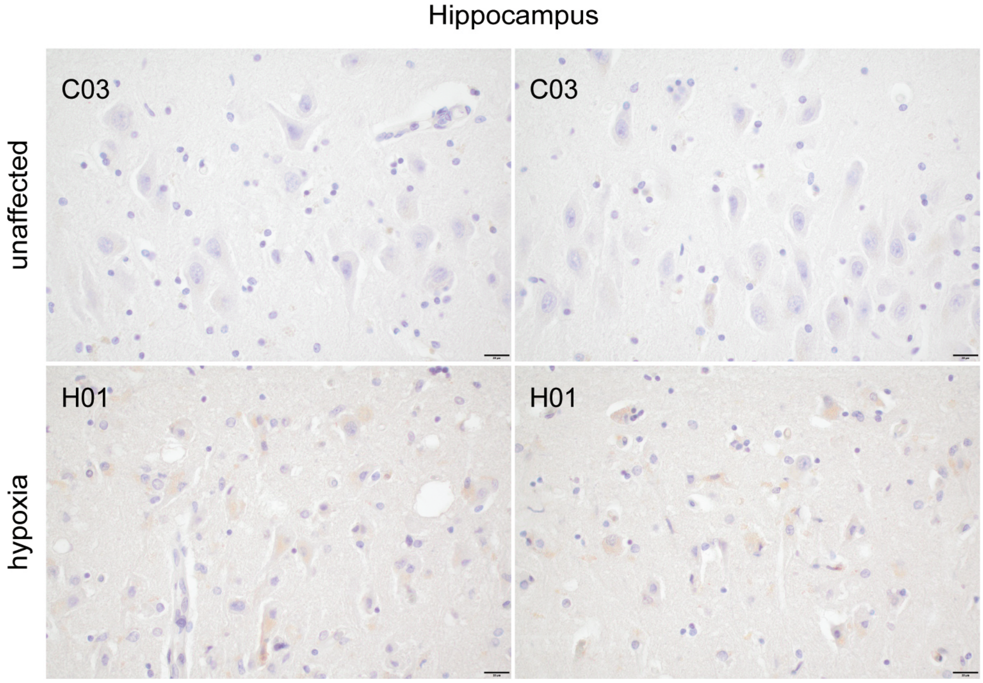
Hippocampus
unaffected
hypoxia
C03
C03
H01
H01

Full-size Western blots, arrows denote the sample areas shown in the figures.

Fig. 2

actin

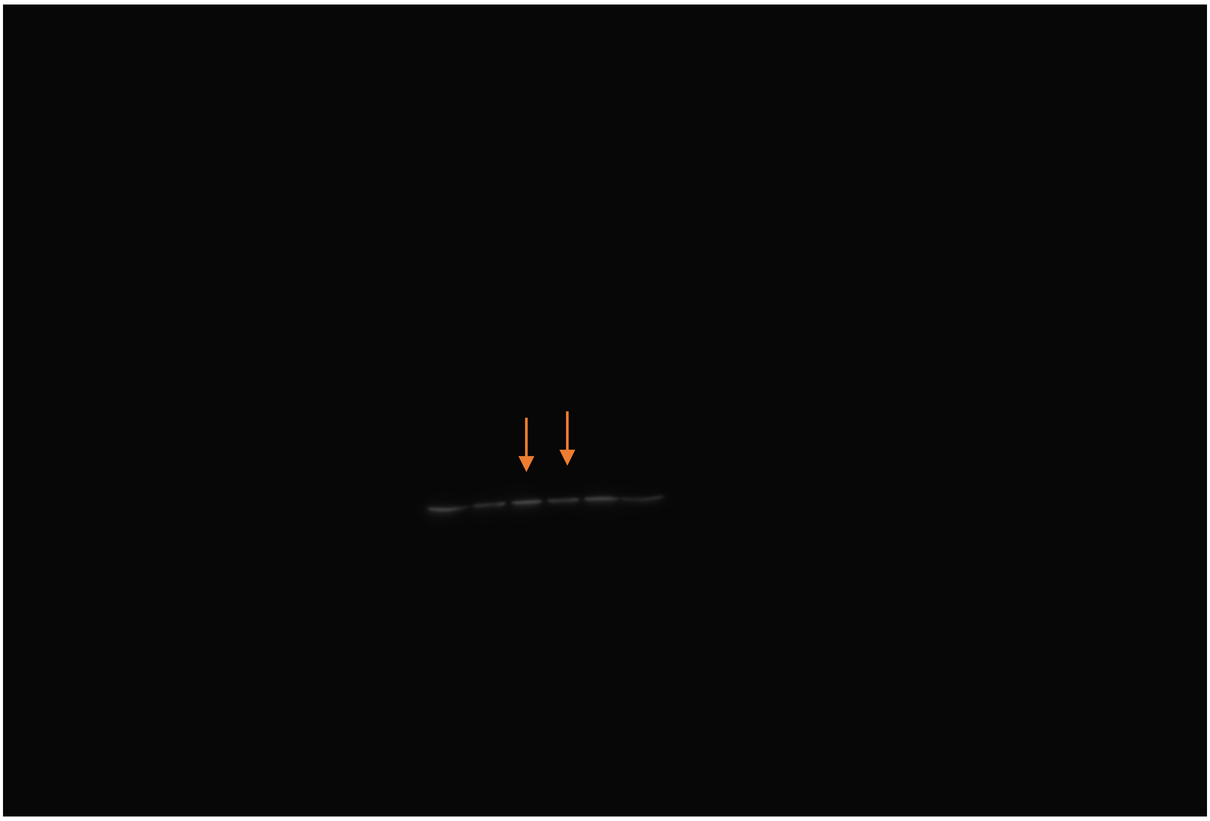

FABP5

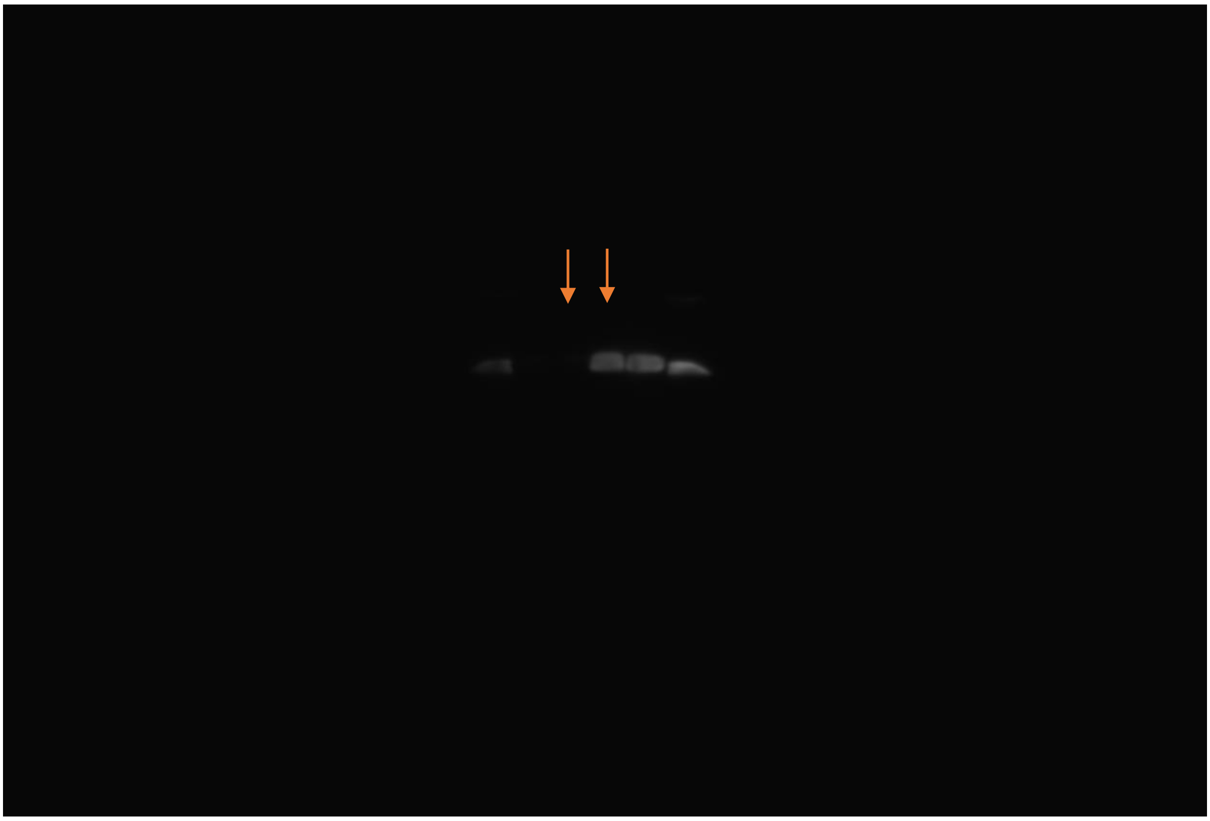

Fig. 4

Cas9

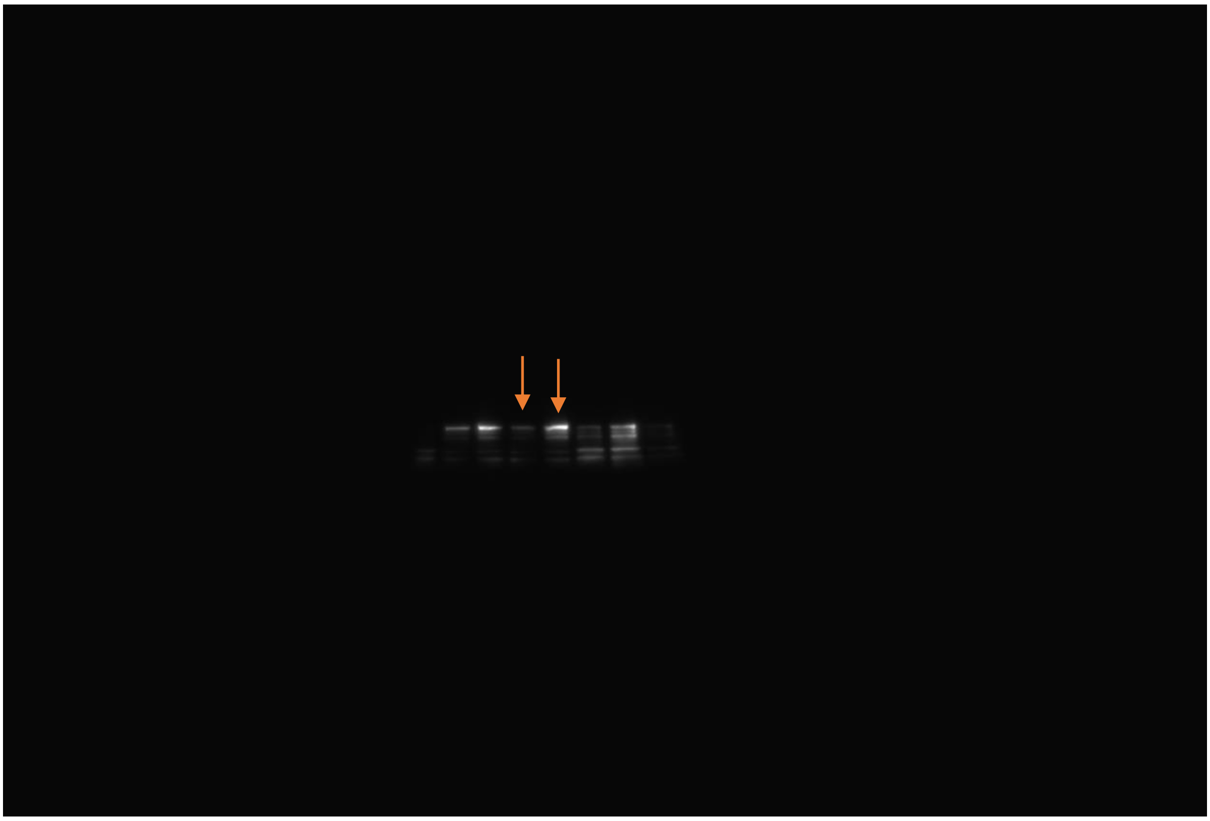

Actin

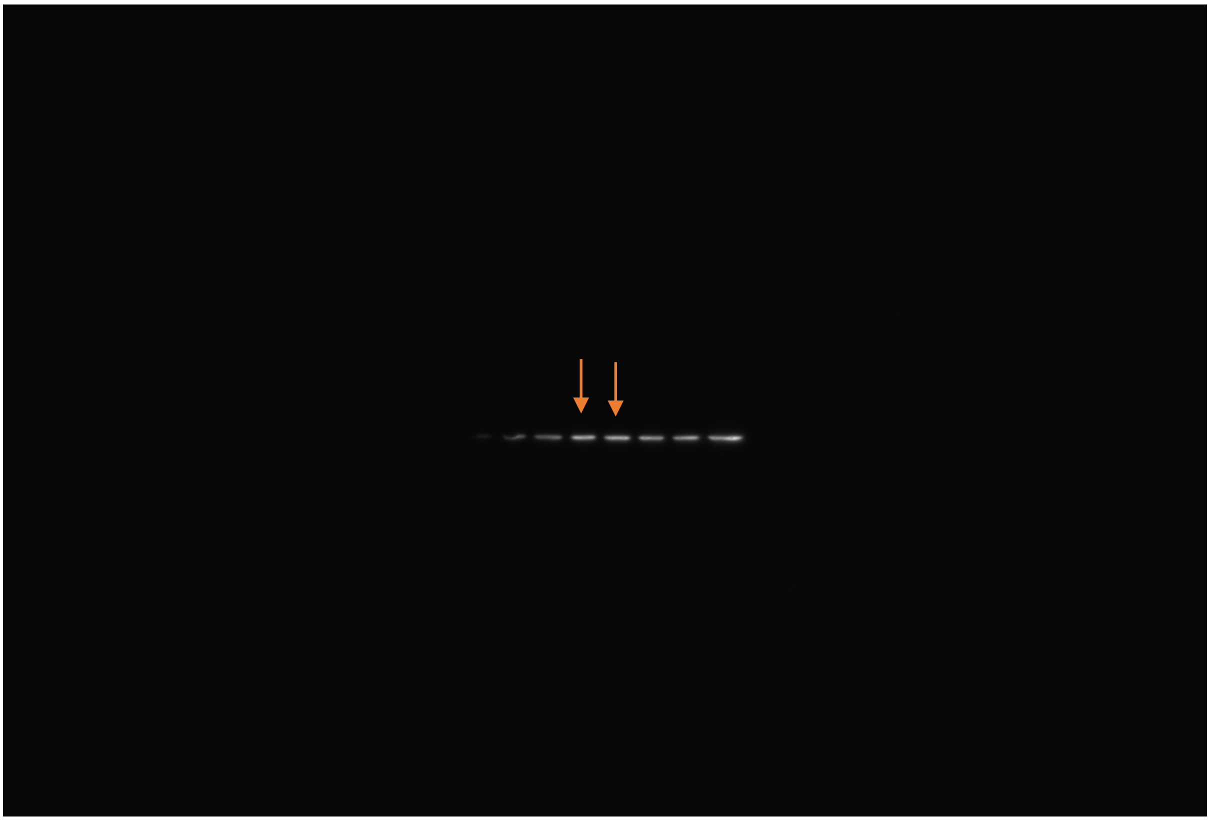

GPX4

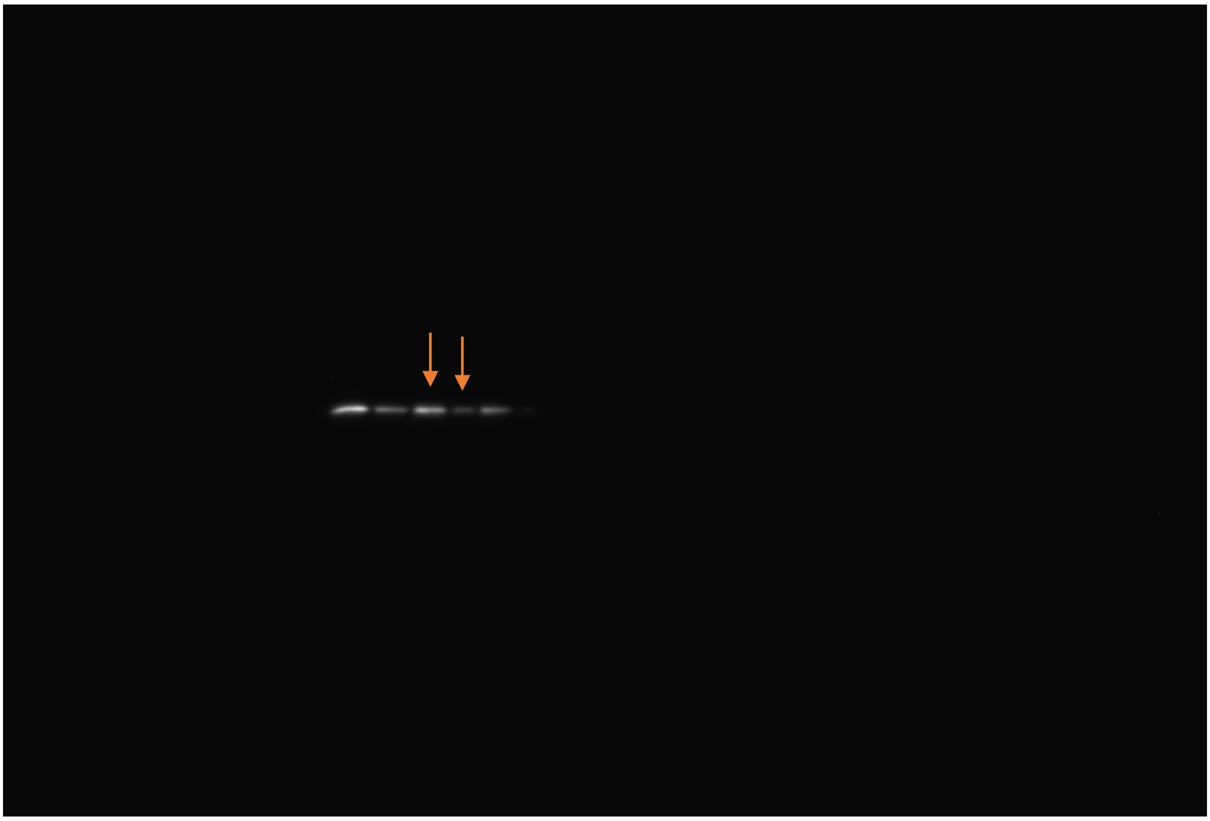

FABP5

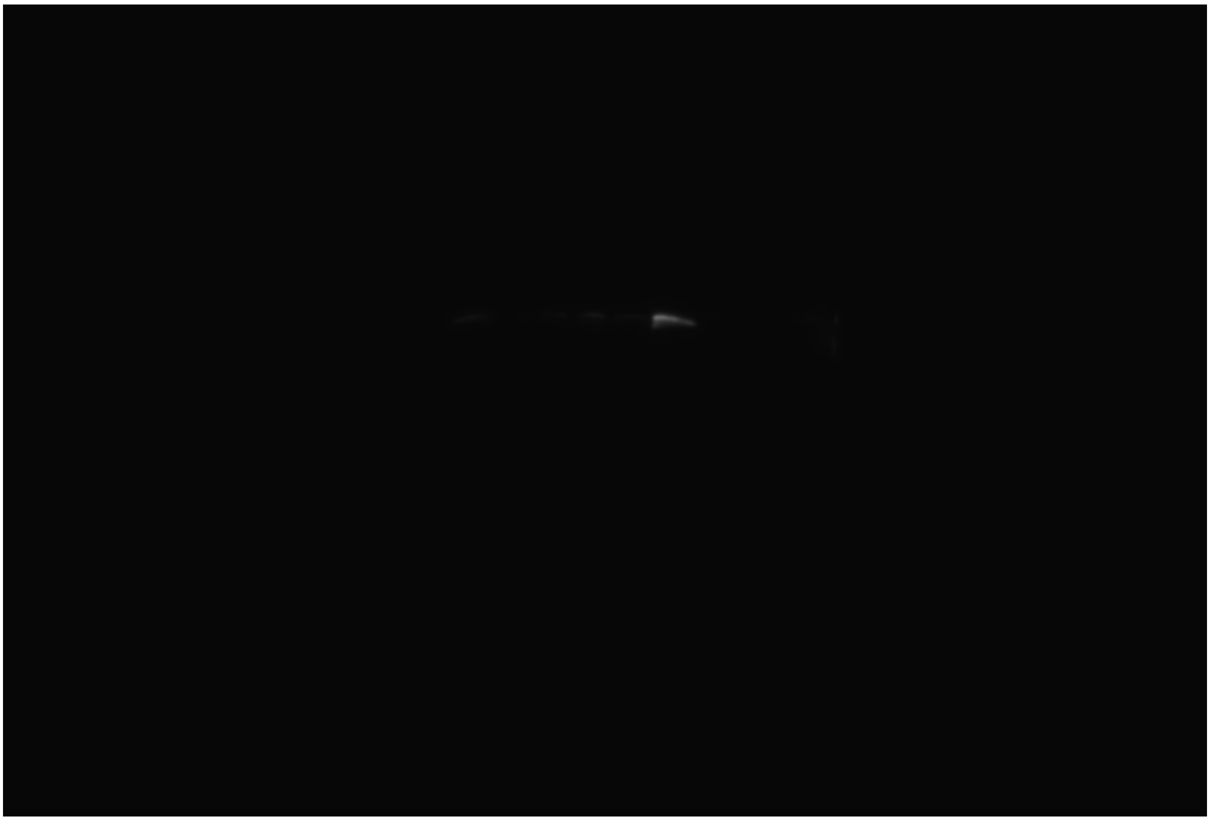

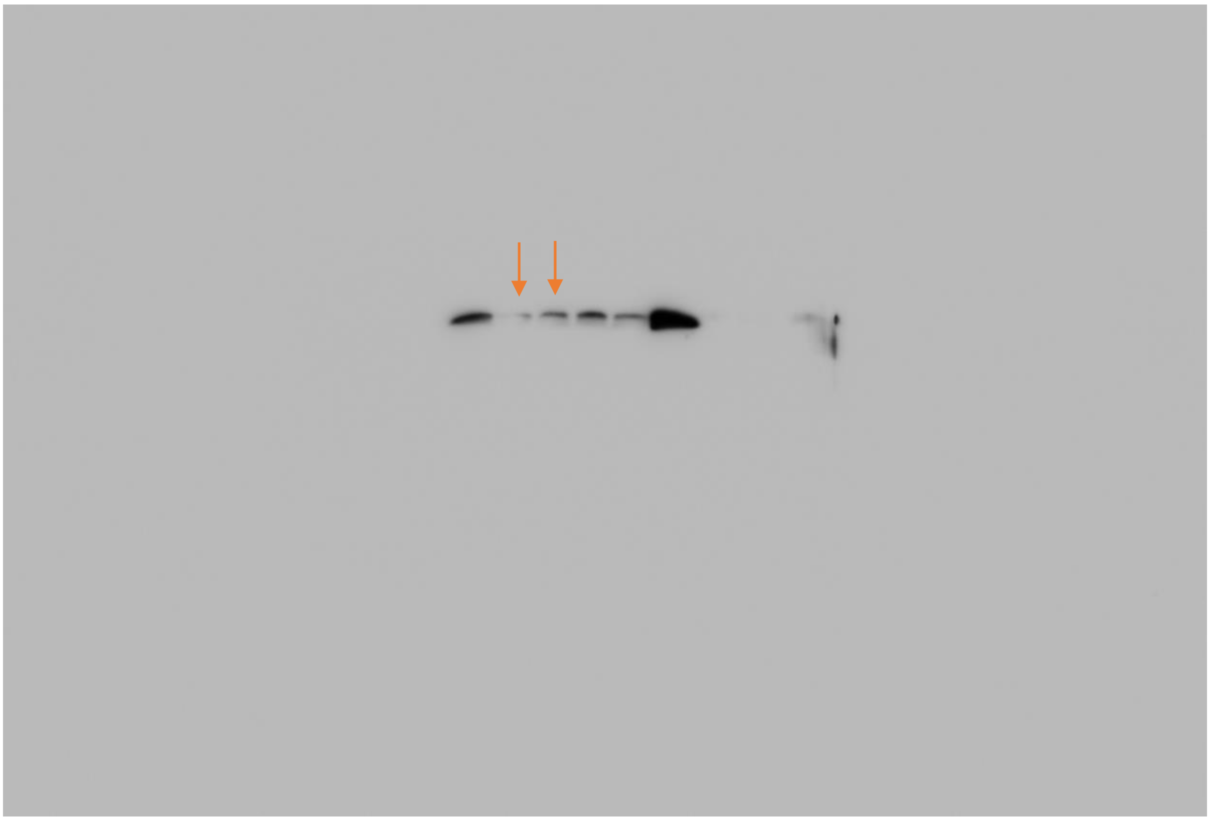

Fig 3

FABP5

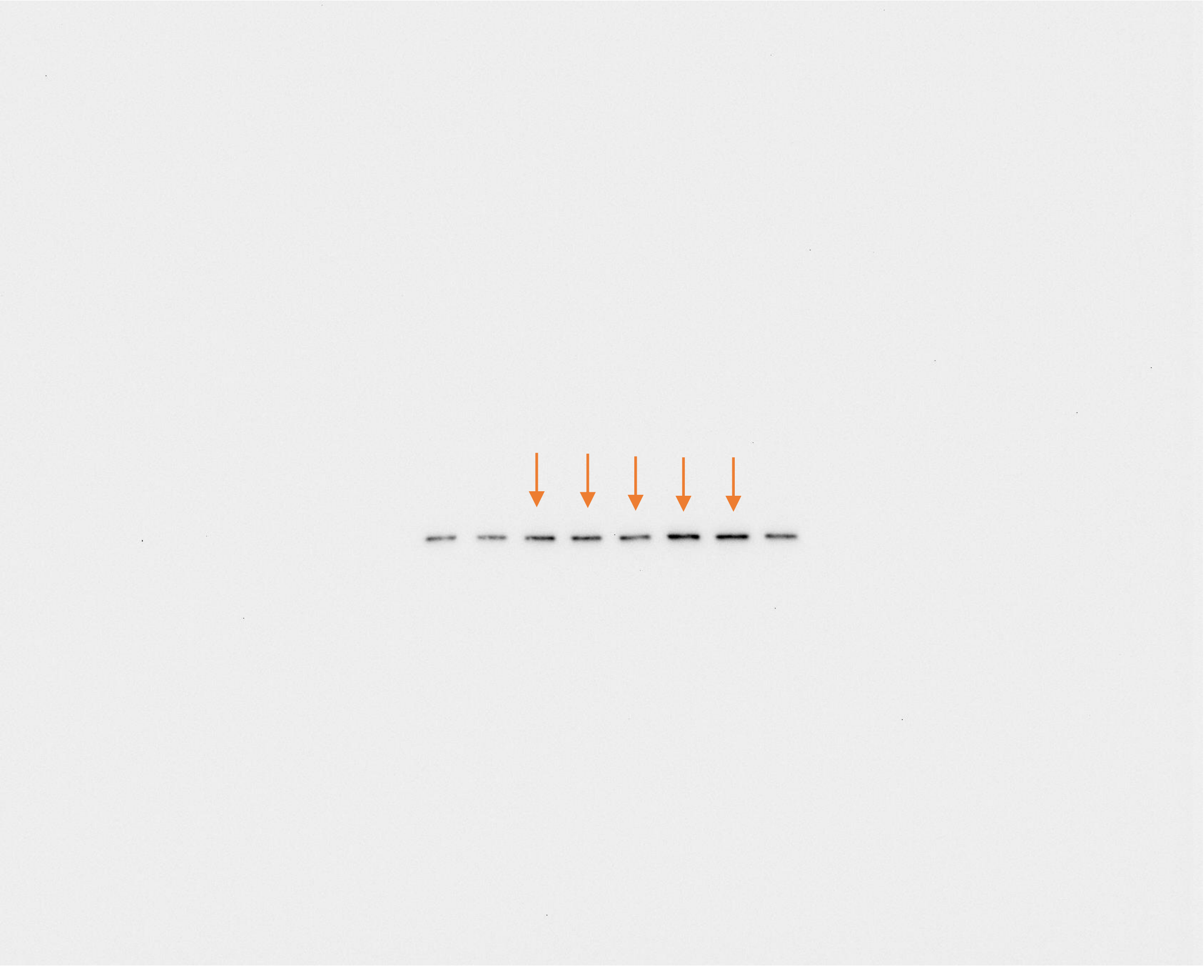

Actin

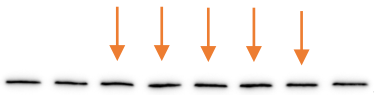

Staurosporine

Actin

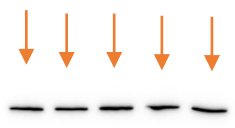

FABP5

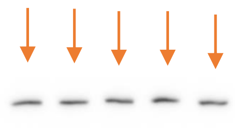

Erastin

Actin

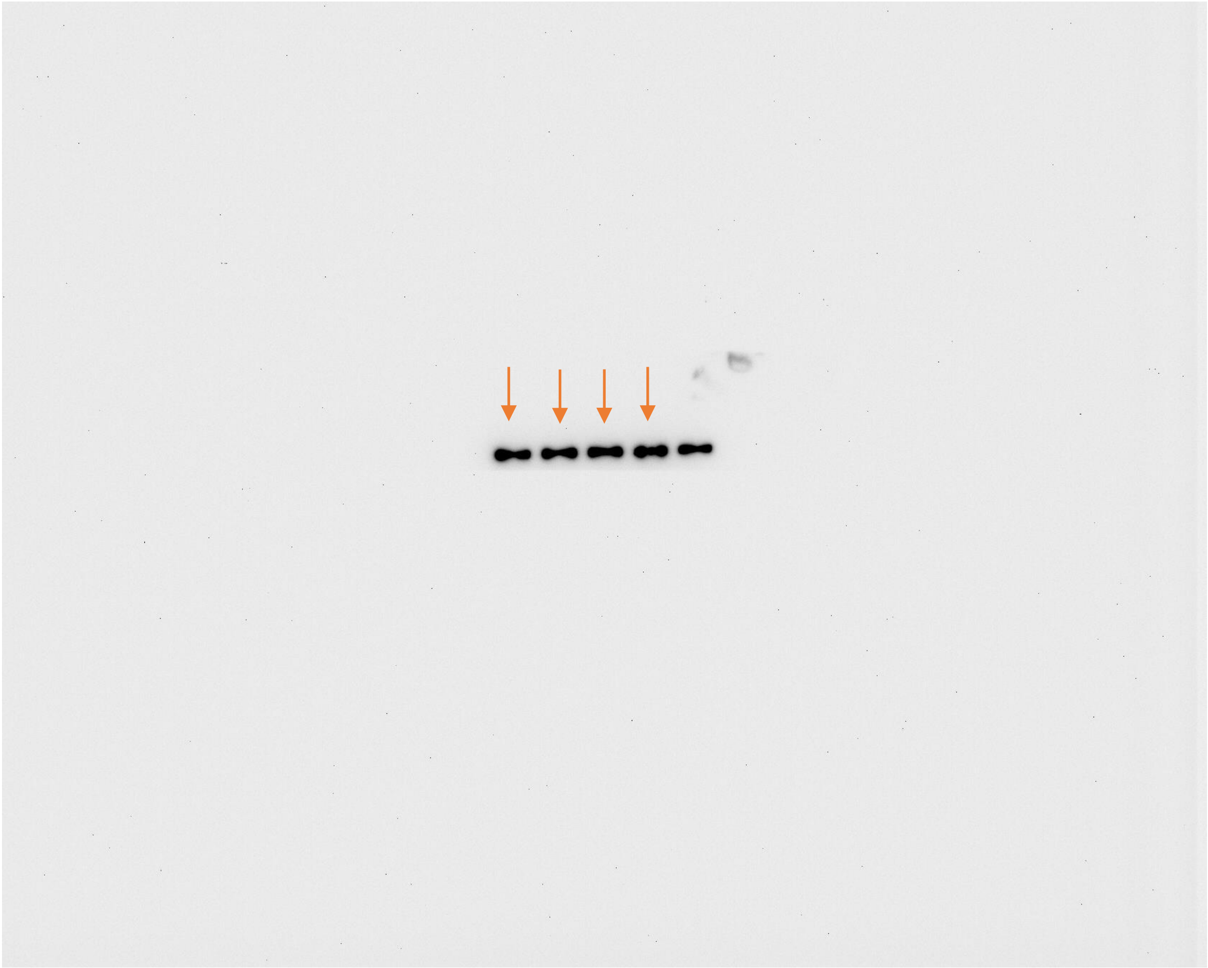

FABP5

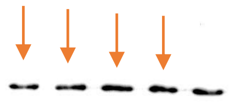

Reagent type (species) or resource	Source	Identifiers	Additional information
Mouse monoclonal anti-CRISPR/Cas9 (7A9-3A3)	Cell signaling	14697	RRID:AB_2800509
Mouse monoclonal anti-Flag	Sigma	F1804	RRID:AB_262044
Rabbit monoclonal anti-Gpx4	Abcam	ab125066	RRID:AB_10973901
Mouse polyclonal anti-LGALS7	R&D Systems	MAB13392	
Rabbit monoclonal anti-ß-Actin (13E5)	Cell signaling	4970	RRID:AB_2223172
Rabbit polyclonal anti-CALML5	Biozol (GeneTex)	GTX119159	RRID:AB_10731097
Rabbit polyclonal anti-CTSV	Elabscience	E-AB-62703	
Rabbit polyclonal anti-FABP5	Cusabio	CSB-PA007946ESR1HU	
Rabbit polyclonal anti-FABP5	Thermo Fisher Scientific	12348-1-AP	RRID:AB_2100341
Rabbit polyclonal anti-FABP5	Thermo Fisher Scientific	PA5-79232	
Rabbit polyclonal anti-S100A14	Biozol (GeneTex)	GTX131119	RRID:AB_2886425
Rabbit polyclonal anti-Cleaved Caspase-3	Cell signaling	Cat# 9661	
Goat anti mouse IgG Cy3	Jackson Immuno	115-165-003	RRID:AB_2338680
Goat anti rabbit IgG Cy2	Jackson Immuno	111-225-003	RRID:AB_2307385
Goat anti rabbit IgG Cy3	Jackson Immuno	111-165-003	RRID:AB_2338000
Mouse anti rabbit IgG Alexa Fluor®594	Biolegend	410407	
phospho-MLKL, Anti-phospho-MLKL (Ser345) Antibody, clone 7C6.1	Sigma	MABC1158	
LIVE/DEAD Fixable Near-IR Dead Cell Stain Kit	Invitrogen	L10119	
XL1-Blue Competent Cells	Agilent	Cat# 200228	
One Shot Stbl3 Chemically Competent E. coli	Thermo Fisher Scientific	Cat# C737303	
Imidazole ketone erastin (IKE)	Stockwell lab		
(1S,3R)-RSL3 (Sig)	Sigma	SML2234	Fig. 3,S2, 4
(1S,3R)-RSL3 (Sto)	Stockwell lab		Fig 1,S1,S3,2
staurosporine	Sigma	Cat# S5921	
BODIPY 493/503	Cayman	121207-31-6	
BODIPY 581/591 C11	Thermo Fisher Scientific	Cat# D3861	
Etoposide	J&K	Cat# 320523	

Colchicine	Serva	Cat# 77120	
Cytochalasin E	Sigma	Cat# C2149	
Cycloheximide (CHX)	Merck	Cat# 239764	
6-Thioguanine (6-TG)	Sigma	Cat# A4882	
Hydrogen Peroxide (H_2O_2)	Sigma	Cat# 31642	
Cisplatin	Sigma	Cat# P4394	
Vinblastine	Sigma	Cat# v1377	
Cyclophosphamide (CP)	J&K	Cat# 419656	
Ferric ammonium citrate	Sigma	F5879-100G	
Formaldehyde solution	Sigma	Cat# 47608	
Erastin	Sigma	Cat# E7781	
2,7-Dichlorodihydrofluorescein diacetate	Cayman	Cay85155	
Human: Calu-1	Stockwell lab		
Human: HCC827, female	ATCC	Cat# CRL-2868	RRID: CVCL_2063
Human: HEK 293T, fetal	ATCC	Cat# CRL-3216	RRID: CVCL_0063
Human: HFF-1, male	ATCC	Cat# SCRC-1041	RRID: CVCL_3285
Human: HT-1080, male	ATCC	Cat# CCL-121	RRID: CVCL_0317
Human: U-138 MG (U138MG), male	ATCC	Cat# HTB-16	RRID: CVCL_0020
Human: SH-SY5Y, female	ATCC	Cat# CRL-2266	RRID: CVCL_0019
psPAX2	Addgene	Cat# 12259	RRID:Addgene_12260
pMD2g	Addgene	Cat# 12260	RRID:Addgene_12260
lentiCRISPRv2	Addgene	Cat# 52961	RRID:Addgene_52961
pLV hU6-sgRNA hUbC-dCas9-KRAB-T2a-Puro	Addgene	Cat# 71236	
pLV hU6 sgRNA hUbC dCas9 KRAB T2a Neo	This paper		
pLVTHM IRES Puro	This paper		
pLVTHM hFABP5 Flag IRES Puro	This paper		
lentiCRISPRv2_hGPX4_sgRNA	This paper		
pLV hU6_hFABP5_sgRNA hUbC dCas9 KRAB T2a Neo	This paper		
DMEM, high glucose, pyruvate, no glutamine	Thermo Fisher Scientific	Cat# 21969035	
RPMI 1640 Medium	Thermo Fisher Scientific	Cat# 21875034	
Fetal Bovine Serum	Thermo Fisher Scientific	Cat# 10270106	
MEM Non-essential Amino Acid Solution (100×)	Sigma	Cat# M7145	
L-Glutamine (200 mM)	Thermo Fisher Scientific	Cat# 25030024	
Penicillin-Streptomycin (10,000 U/mL)	Thermo Fisher Scientific	Cat# 15140122	
Puromycin dihydrochloride	Sigma	Cat# P9620; CAS: 58-58-2	
X-tremeGENE HP DNA Transfection Reagent	Sigma	Cat# 6366244001	
Accutase solution	Sigma	Cat# A6964	
BCA kit	Thermo Fisher Scientific	Cat# 22663, Cat#22660	

Skim milk powder	Sigma	Cat# 70166-500G	
ECL	Bio-RAD	Cat# 1705060	
Pierce™ Cell Surface Protein Isolation Kit	Thermo Fisher Scientific	Cat# 89881	
GraphPad Prism	GraphPad Software	www.graphpad.com	
FlowJo 10	FlowJo LLC	www.flowjo.com	
Columbus 2.9.1.532	PerkinElmer		
Harmony software			

CRISPR guide sequences and Primers

**CRISPR guide sequences**	**SOURCE**	**IDENTIFIER**
Human *GPX4* KO guide 1: CACCGACCAACGTGGCCTCCCAGTG	This paper	N/A
Human *GPX4* KO guide 2: AAACCACTGGGAGGCCACGTTGGTC	This paper	N/A
Human *FABP5* Knockdown guide 1 CGAGAGGCCGTCGCGTACCC	This paper	N/A
Human *FABP5* Knockdown guide 2 GCTAACCAGCTCCTCTTGCC	This paper	N/A
Human *FABP5* Knockdown guide 3 ACGCCGGCGCCTGCACGTTT	This paper	N/A
**Cloning primers**		
Human *FABP5* amplification forward primer: ACGAGACTAGCCTCGAGGTTTAAACGCCACCATGGCCACA GTTCAGCAG CTGGA	This paper	N/A
Human *FABP5* amplification reverse primer: TGCCTTCACAAAGATCCTCATATGTCACTTATCGTCATCGT CTTTGTAATC TTCTACTTTTTCATAGATCCGAG	This paper	N/A
**qPCR primers**		
Human *CALML5* qPCR forward primer: gccctgcagtggaatgag	This paper	N/A
Human *CALML5* qPCR reverse primer: ttcatggaactcggcagtc	This paper	N/A
Human *FABP5* qPCR forward primer: gcagacccctctctgcac	This paper	N/A
Human *FABP5* qPCR reverse primer:	This paper	N/A

tcgcaaagctattcccactc		
Human *LGALS7* qPCR forward primer:<br>ggcttggttcctcccaat	This paper	N/A
Human *LGALS7* qPCR reverse primer:<br>ccttgctccttgctgttga	This paper	N/A
Human *S100A14* qPCR forward primer:<br>cttctgagctacgggacctg	This paper	N/A
Human *S100A14* qPCR reverse primer:<br>ttctcttccaggccacagtt	This paper	N/A
Human *CTSV* qPCR forward primer:<br>ggcaacacacagaagattatatgg	This paper	N/A
Human *CTSV* qPCR reverse primer:<br>tcattttcatattcttttcccaca	This paper	N/A
